# A Comprehensive Survey on Deep Learning-Based LoRa Radio Frequency Fingerprinting Identification

**DOI:** 10.3390/s24134411

**Published:** 2024-07-08

**Authors:** Aqeel Ahmed, Bruno Quoitin, Alexander Gros, Veronique Moeyaert

**Affiliations:** 1 Department of Computer Science, Faculty of Science, University of Mons, Av. du Champ de Mars, 7000 Mons, Belgium; aqeel.ahmed@umons.ac.be; 2 Department of Electromagnetism and Telecommunication, Faculty of Engineering, University of Mons, 31 Boulevard Dolez, 7000 Mons, Belgium; alexander.gros@umons.ac.be (A.G.); veronique.moeyaert@umons.ac.be (V.M.)

**Keywords:** LoRaWAN, RF fingerprinting, device identification, deep learning, LoRa PHY, wireless security

## Abstract

LoRa enables long-range communication for Internet of Things (IoT) devices, especially those with limited resources and low power requirements. Consequently, LoRa has emerged as a popular choice for numerous IoT applications. However, the security of LoRa devices is one of the major concerns that requires attention. Existing device identification mechanisms use cryptography which has two major issues: (1) cryptography is hard on the device resources and (2) physical attacks might prevent them from being effective. Deep learning-based radio frequency fingerprinting identification (RFFI) is emerging as a key candidate for device identification using hardware-intrinsic features. In this paper, we present a comprehensive survey of the state of the art in the area of deep learning-based radio frequency fingerprinting identification for LoRa devices. We discuss various categories of radio frequency fingerprinting techniques along with hardware imperfections that can be exploited to identify an emitter. Furthermore, we describe different deep learning algorithms implemented for the task of LoRa device classification and summarize the main approaches and results. We discuss several representations of the LoRa signal used as input to deep learning models. Additionally, we provide a thorough review of all the LoRa RF signal datasets used in the literature and summarize details about the hardware used, the type of signals collected, the features provided, availability, and size. Finally, we conclude this paper by discussing the existing challenges in deep learning-based LoRa device identification and also envisage future research directions and opportunities.

## 1. Introduction

Our reliance on the Internet of Things (IoT) is increasing progressively as we are moving towards a smart planet enabled by smart technologies such as smart cities, smart homes, smart agriculture, smart healthcare, smart mobility, and smart logistics. The exponential growth of the IoT has already transformed various sectors of our lives by leveraging its ability to connect objects and automate tasks. This surge is exponential, and there are already more Internet-connected objects than humans on the planet. At the current pace of connecting objects to the Internet, it is expected to reach the 30 × $10^{9}$ mark by 2030 [1].

The IoT devices are mostly wirelessly connected using various protocols, each with unique characteristics, i.e., data rate, range of communication, and energy consumption [2]. Among the short-range wireless protocols: Bluetooth [3], Zigbee [4], Bluetooth Low Energy (BLE) [5], and WiFi [6] are popular and vastly used. However, WiFi provides higher data rates compared to others but also consumes more energy. On the other hand, Low-Power Wide Area Network (LPWAN) [7] is an emerging class of wireless communication technologies suitable for IoT applications that require long-range communication at low data rates. Sigfox [8], Narrow-Band IoT (NB-IoT) [9] and LoRaWAN [10] are popular LPWAN standards. Among them, LoRaWAN is a promising network architecture that has gained massive popularity in the past few years. LoRaWAN is built on top of the LoRa technology [11] which defines the physical layer. Although these LPWAN standards offer multi-fold advantages in terms of low cost, low energy, and distant communication, the security of these protocols is a huge challenge. Despite the fact that the communication in LoRaWAN is encrypted, device identification remains vulnerable to security attacks such as impersonation and provides a ground for malicious actors to exploit the loopholes.

To address the challenging security issues of the LoRa protocol, deep learning-based radio frequency fingerprinting identification (RFFI) has gained significant attention among researchers and is now widely accepted as a trending subject. Here, *fingerprints* refer to the entangled features of electronic hardware originating from the manufacturing process. Even with the latest advancements in manufacturing technology, the dissimilarity of the analog components of radio transmitters still creates variations in the signal waveform. *Radio frequency fingerprinting* is the process of extracting those features from the radio signal of the device with hardware imperfections that make the transmitter distinguishable among others even if they are from the same model and manufacturer [12]. The impairments in the wireless signal are device-intrinsic, unique, and hard to tamper with. Although these unusual components in the waveform do not significantly affect the communication link, they are powerful enough to be used as radio emitter identifiers. The traditional techniques of extracting features from radio signals are not only complicated to derive and time-consuming but require strong domain expertise [12].

Deep learning (DL) algorithms have proven effective at extracting features from wireless signals that originated due to device imperfections. In this survey article, we specifically focus on LoRa protocol and comprehensively review the state of the art in deep learning-based LoRa device identification using radio frequency fingerprints (RFFs).

### 1.1. Motivation

There are several survey papers published in the area of radio frequency fingerprinting methods for various technologies [13,14,15,16,17,18,19,20,21,22]. Jagannath et al. [13] provided a comprehensive review of the traditional RFF method and also partially discussed DL-based RFF techniques. In [14], RFF techniques are surveyed for IoT device fingerprinting using features from the network traces, MAC frame, and radio features. Alshammri et al. [15] reviewed the security attacks on radio frequency identification systems and categorized several attacks, and also provided a discussion on prevention mechanisms. In [16], several RF fingerprints for secure device authentication are described. Gorbani et al. [17] provided a detailed survey on profiling, fingerprinting, and identification methods for IoT devices. Soltanieh et al. [18] presented a discussion on transient and steady-state RF fingerprinting techniques along with various RF signal features that can be used to identify a radio emitter. In [19,20], transient and steady-state RFF methods along with DL applications for radio emitter identification are discussed. Sun et al. [21] and Safi et al. [22] partially discuss the DL-based RFFI and LoRa signal identification. One of the most recent reviews is published by Xie et al. [23], which comprehensively reviews the state of the art in radio frequency fingerprinting for IoT systems.

As mentioned, the existing literature is focused more on general approaches of RFFI for different technologies. The only specific and IoT-targeted review of the RFFI technique was recently published by Xie et al. [23]. However, there is no study targeting specifically DL-based RFFI for the LoRa system comprehensively and suggests future research directions. In this survey, we focus on various aspects of LoRa technology including the background on CSS modulation, LoRa-specific hardware impairments, and the proposed RFFI method. We also analyze the LoRa RFFI datasets in-depth and discuss their limitations.

### 1.2. Research Contribution

In this paper, we comprehensively review the state of the art which particularly targets deep learning-based radio frequency fingerprinting identification for LoRa devices. The core contribution of the paper is the critical analysis of the existing literature in this particular domain. We summarize our contribution as follows:

- We focus on LoRa technology and comprehensively review the existing literature published in the domain of deep learning-based LoRa radio frequency fingerprint identification. The existing surveys on radio frequency fingerprinting are not domain-specific and not as comprehensive as this paper.
- We discuss several RF fingerprinting methods and their applications with pros and cons.
- We present a comprehensive discussion on different hardware impairment types and their modeling in typical radio frequency transceivers with an additional focus on LoRa-specific impairments.
- We summarize all the deep learning-based LoRa RFFI systems in terms of approach adaptation, model selection, LoRa signal representation, and performance.
- We analyze all the LoRa RFF datasets used in the literature and present a comprehensive tabular summary of all the characteristics of these datasets.
- We systematically present the challenges in the state of the art at different levels and also envisage the future research directions.

### 1.3. Organization of the Survey

**Figure 1:**
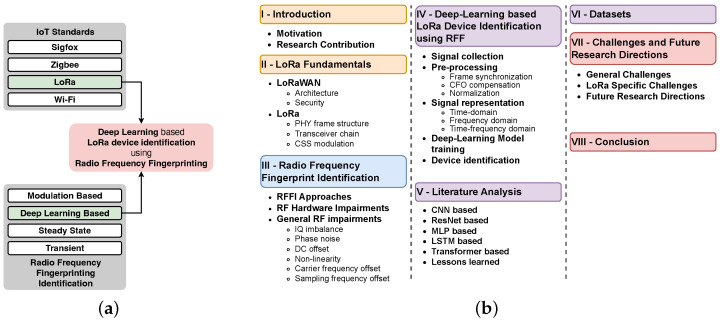
Scope (**a**) and organization (**b**) of the survey.

This article aims to provide a detailed analysis of the literature published in the area of deep learning-based LoRa device identification using radio frequency fingerprinting. Figure 1 shows the scope and organization of this survey. In Section 2, we provide a description of the LoRaWAN fundamentals that cover LoRaWAN architecture, security aspect, and the LoRa radio communication technology, with a focus on LoRa CSS modulation and transceiver chain. Section 3 is dedicated to radio frequency fingerprint identification (RFFI) and the hardware impairments in RF transceivers. Section 4 describes the deep learning-based LoRa RFFI pipeline. We provide a critical analysis of the literature in Section 5, and we discuss the LoRa RFFI datasets in Section 6. The paper is further extended with Section 7 which covers various challenges and future research directions in the domain. Finally, we conclude our work in Section 8.

## 2. LoRa Fundamentals

This section describes the basic principles of LoRa, covering the LoRaWAN architecture and security mechanisms, as well as details on LoRa Chirp Spread Spectrum (CSS) modulation. We extend this section with details of the LoRa PHY frame structure and end the section with an explanation of the LoRa transceiver chain.

### 2.1. LoRaWAN Architecture

**Figure 2:**
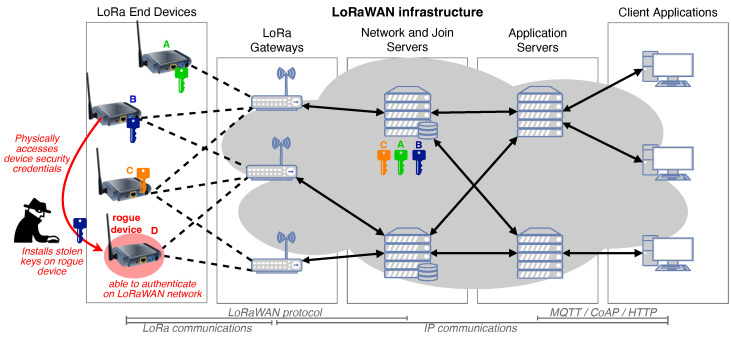
LoRaWAN architecture and impersonation attack.

LoRaWAN is a wide area network infrastructure that enables LoRa end devices to be connected to remote applications via the Internet. A typical LoRaWAN architecture is shown in Figure 2. A LoRaWAN network is created in the star-of-stars topology, where gateways deployed at the edge of the network provide connectivity to the end devices. Communications between end devices and gateways make use of the LoRa radio frequency (RF) communication technology. A LoRa end device therefore includes a LoRa transceiver that can transmit and receive this RF signal. Each LoRa transceiver can broadcast its signal to all the gateways in its vicinity. The core of the LoRaWAN network is made up of different kinds of servers. Network and join servers are responsible for authenticating devices and routing their messages, while application servers make these messages available to remote client applications. Communications between LoRaWAN gateways, servers, and client applications are based on an IP backhaul using various technologies such as Ethernet, Wi-Fi, or LTE links.

LoRaWAN allows bi-directional communications: messages going from the end devices to the gateways are called up-link messages while the others are called down-link messages. It should be noted that end devices do not communicate directly with each other. The typical use case for LoRaWAN devices is to collect data that are then sent through the LoRaWAN architecture. Up-link messages are therefore more frequent than down-link messages.

To help optimize the energy consumption and the time on air of the signal, LoRaWAN supports an Adaptive Data Rate (ADR) mechanism. It allows dynamic control of the transmission parameters of the end devices such as the Spreading Factor (SF), Bandwidth, and transmission power.

### 2.2. LoRaWAN Security

The LoRaWAN specification defines two methods for an end device to join the network, namely *Activation By Personalization* (ABP) and *Over The Air Activation* (OTAA). While both of them have pros and cons, the latter is considered more flexible and more secure [24]. LoRaWAN ensures end-to-end security using the Advanced Encryption Standard (AES-128) [25]. Each end device has its own Application Key (AppKey) that is pre-shared with the LoRaWAN network (or join) server. Two session keys are derived from the AppKey when the end device joins the LoRaWAN network. The Network Session Key (NwkSKey) is used to create and validate the integrity of messages, while the Application Session Key (AppSKey) is used for data encryption [26].

The end device authentication is performed by calculating a Message Integrity Code (MIC) on its device identifier (DevEUI) and a nonce using its AppKey. The network/join server calculates the same MIC using the AppKey associated with the DevEUI in its database. If the two MICs match, the end device is authorized and receives as a response the necessary cryptographic material to derive its AppSKey and NwkSKey.

Despite the end-to-end security provided by LoRaWAN, the end devices are still vulnerable to security attacks such as wormhole attacks [26], Denial of Service (DoS) [24], replay attacks [27], and jamming [28]. The LoRa end devices store the security credentials in their non-volatile memory; hence, if they are physically accessed, they can be tampered with and the security keys can be extracted, as shown in Figure 2. Once compromised, the rogue node can imitate the behavior of legitimate devices within the network and carry out various cyber attacks. The identification of rogue devices that possess authentic security keys is impossible using existing cryptographic security techniques. The heavy cryptographic methods will require additional resources to be applied, as the current processes are resource-intensive, whereas for the DL-based RFFI system, the end device does not need to provide any additional resources as DL-based RFFI will be deployed on LoRa gateways. Additionally, DL approaches also eliminate the challenge of manual feature extraction from RFFs which is a difficult and cumbersome process.

### 2.3. LoRa CSS Modulation

LoRa [29] is a physical layer RF communication technology invented by the French company Cycleo, acquired by Semtech in 2012. It is based on Chirp Spread Spectrum (CSS) modulation, a technique used for radar communications in the past because of its anti-interference and long-range capabilities. Sending data with LoRa provides multi-fold advantages including receiver design simplicity, bandwidth scalability, low power consumption, and robustness against interference, multi-path fading, and Doppler effects. The main factor that makes the LoRa robust against interference is the chirp frequency variation over time during the modulation process. The spreading of the data signal across the wide band makes it less susceptible to noise and interference. In addition, the LoRa receiver applies the matched filtering during the dechirping process. This allows the LoRa receiver to demodulate the symbols even in the presence of a low signal-to-noise ratio.

LoRa operates in a license-free Industrial, Scientific, and Medical (ISM) band of frequencies. The operating frequency ranges of LoRa change regionally. It operates at 433 MHz and 868 MHz in Europe, at 915 MHz in North America and at 923 MHz in Asia and Australia. In addition, LoRa operations in 2.4 GHz band have also been proposed to enable the worldwide frequency channels.

**Figure 3:**
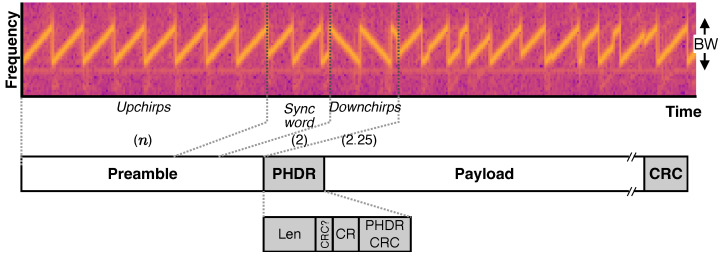
Spectrogram and structure of a LoRa frame. The color intensity in the spectrogram shows the power in that particular instance of time and frequency, with yellow color representing higher power than pink color. The preamble at the beginning of the frame is made up of a sequence of unmodulated up-chirps terminated by two and a quarter unmodulated down-chirps.

CSS modulation uses chirps to transmit data. A *chirp* is a signal whose frequency varies continuously and linearly with time. A chirp with increasing frequency is called an *up-chirp*, while the opposite is called a *down-chirp*. Figure 3 shows a LoRa signal exhibiting up- and down-chirps. The frequency of the chirp signal is swept over the entire bandwidth (BW).

The detailed operations of the LoRa physical layer are not disclosed by Semtech. However, some details are revealed in the patent [30], specifically the modulation technique. The missing details were obtained by reverse engineering [31].

#### 2.3.1. LoRa PHY Frame Structure

The LoRa PHY frame structure as presented by [31], consists of various fields. We depict the frame structure in Figure 3 and briefly describe each field below.

- **Preamble**: The preamble is the initial part of the LoRa PHY frame. It is used for the synchronization of the transmitter and receiver. The preamble starts with a sequence of *n* up-chirps (usually n=8), followed by two sync word chirps, and ends with two and a quarter of down-chirps.
- **Physical Header (PHDR)**: This field contains information related to payload size (Len), coding rate (CR), presence of a cyclic redundancy check trailer (CRC?), and a PHDR-specific CRC (PHDR CRC).
- **Payload**: This field carries the upper layer frames, such as LoRaWAN MAC frames. The maximum payload size is up to 255 bytes but can be further limited depending on the data rate and region of operation.
- **CRC**: This field carries a 16-bit CRC that covers the payload. The presence of this field depends on the bit CRC in the PHDR.

LoRa frames may optionally be sent in *implicit mode* when all the frame parameters are well-known. In this case, the PHDR field can be elided. Implicit mode is used for example by LoRaWAN beacons which have a fixed length.

#### 2.3.2. LoRa Transceiver Chain

**Figure 4:**
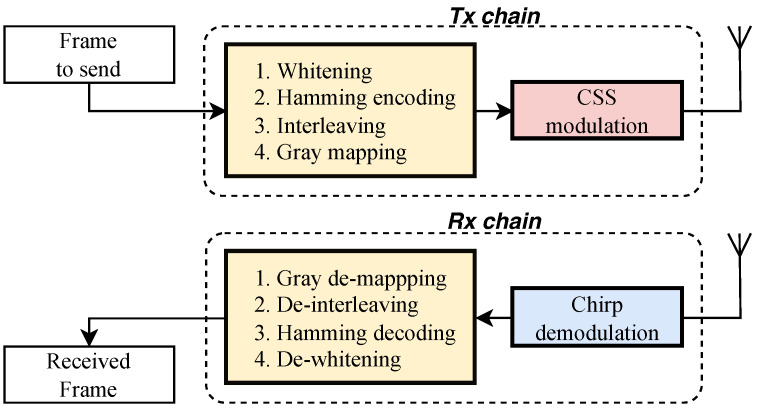
A typical LoRa transceiver chain (adapted from [31]).

The LoRa transceiver chain refers to all the elements involved in transmitting a LoRa frame until it is transformed into an RF signal. Figure 4 shows the main blocks of a LoRa transceiver for both directions of operation. We first briefly discuss operations that are carried on the frame bits such as whitening and Hamming coding. Then, since our focus is on radio frequency fingerprinting, we proceed with a more in-depth discussion of CSS modulation.

#### 2.3.3. Frame Coding

- The first step in the LoRa transceiver chain is ***whitening*** which involves XORing the information bits with a pseudo-random binary sequence. Bit correlation is introduced in transmission by the channel encoder which adds redundancy bits. This may be problematic in the overall transmission chain. Hence, the whitening process is required. Because the original Semtech whitening sequence is not known, different whitening sequences have been used in LoRa PHY open-source implementations [31,32,33].
- After whitening and the insertion of the PHDR and CRC, the next stage is ***Hamming encoding*** which enables robust and error-free transmission. According to [31], LoRa uses a variation of the original Hamming code to detect and even correct errors in the data. The LoRa parameter *Code Rate* (CR) controls the amount of redundancy introduced.
- The Hamming code stage is followed by ***interleaving*** which is crucial to minimize the impact of burst errors caused by noise or fading of the signal. It achieves the shuffle of bits over multiple code words. The process of interleaving in LoRa is detailed in [34].
- The final stage before chirp modulation is ***Gray mapping***. Using the Gray code, two successive symbols differ by only one bit. Mapping them to binary sequences using Gray code improves the performance of the error correction technique and lowers the bit error rate.

#### 2.3.4. Chirp Modulation

**Figure 5:**
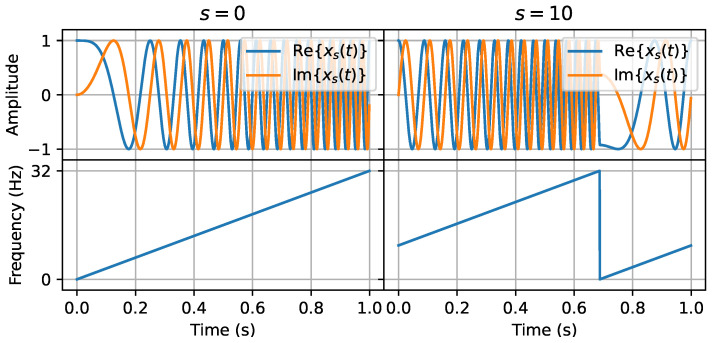
Example CSS Modulated Symbols (BW=32 Hz and SF=5).

LoRa CSS modulation [31,35,36] relies on chirps to carry data. The waveforms of these chirps depend on two important parameters. First, the channel bandwidth (B) is the amount of frequency spectrum that is swept by a chirp. Typical values for B are 125 KHz and 250 KHz. Second, the *spreading factor* (SF), is the number of bits carried per LoRa symbol. It ranges between 7 and 12. The duration $T_{c}$ of a chirp is a function of these two parameters: $T_{c}=2^{\mathrm{SF}}/B$. The left side of Figure 5 shows the waveform of an up-chirp, using B = 32 Hz and SF = 5. As a result, its duration is 1 s.

When the sampling interval is equal to 1/B, as proposed in the LoRa patent, the discrete-time waveform of an unmodulated up-chirp is well described by Equation (1). In this case, there are $N=2^{\mathrm{SF}}$ samples per chirp and 0≤k<N.

(1) $$x_{0}\left[k\right]=e^{j\pi \frac{k^{2}}{N}}$$

LoRa transmits a sequence of SF bits per up-chirp. This sequence of bits is mapped to a LoRa baseband symbol *s* which is a value in the range 0 to $2^{\mathrm{SF}}-1$. To carry a symbol *s*, an up-chirp is circularly time-shifted proportionally to *s*. The frequency of a modulated up-chirp starts at $\frac{sB}{N}$ and linearly increases at a rate of $\frac{B}{T_{c}}$ until it reaches B. It then folds down to 0 and the frequency again starts to increase until it reaches the initial point. This behavior is illustrated for symbol s=10 on the right side of Figure 5. The up-chirp starts with frequency $\frac{sB}{N}=10$ Hz, increases linearly until it reaches 32 Hz then drops steeply down to 0 Hz and continues to increase until it reaches the starting frequency. The discrete-time version of the baseband LoRa waveform $x_{s}\left[n\right]$ as presented in [31], is given by Equation (2).

(2) $$x_{s}\left[k\right]=e^{j2\pi \left(\frac{k^{2}}{2N}+\frac{sk}{N}\right)}$$

#### 2.3.5. Chirp Demodulation

When the signal is transmitted over an AWGN channel, the received signal can be written as (3), where z[k] is the complex value Gaussian noise with variance $\sigma^{2}$ and zero mean.

(3) $$y\left[k\right]=x_{s}\left[k\right]+z\left[k\right]$$

In a typical non-coherent LoRa receiver, the symbols are demodulated by applying matched filters with reference symbols and then retrieved by maximum likelihood. However, this process is computationally expensive as it requires $2^{SF}$ convolutions. An efficient implementation is the *dechirping* process, i.e., multiplying the samples of the received modulated symbol y=(y[0],⋯,y[N−1]) with the complex conjugate of a reference up-chirp $x_{0}^{*}=\left(x_{0}^{*}\left[0\right],\cdots ,x_{0}^{*}\left[N-1\right]\right)$, as shown in Equation (4). Herem ⊙ denotes the element-wise product of two vectors. This approach assumes the receiver is phase-synchronized, using the preamble of the incoming frame.

(4) $$y^{\prime }=y⊙x_{0}^{*}$$

Then, the symbols are retrieved by applying the Discrete Fourier Transform (DFT), $Y=\mathrm{DFT}(y^{\prime })$. The received symbol corresponds to the frequency bin with the highest magnitude, that is $\hat{s}=\arg\;\max_{0\leq k<N}\left|Y[k]\right|$.

After CSS demodulation, the receiver performs Gray de-mapping, de-interleaving, Hamming decoding, and de-whitening to retrieve the data bits of the received LoRa frame.

## 3. RF Fingerprinting Identification

Even though manufacturing technology has advanced in recent years, RF transceivers can still have hardware impairments due to analog components such as filters, oscillators, mixers, power amplifiers, and even antennas [37,38,39]. The presence of these impairments leads to inherent variations in the received RF waveform. While these variations may not significantly impact overall communication performance, they are consistent, distinctive, and difficult to manipulate. Hence, they can serve as reliable device identifiers. We discuss traditional RFFI approaches in Section 3.1. Later we briefly describe RF hardware impairments in Section 3.2.

### 3.1. RFFI Approaches

Radio frequency fingerprinting identification (RFFI) is now a well-established concept in which unique features of an RF transmitter are extracted from its waveform and used as an identifier. It is a form of signal intelligence that enables device identification and authentication in wireless networks [40]. These features are generated due to the specific imperfection in the RF devices. The popularity of RFFI is growing as a physical layer security method for resource-constrained IoT devices as RFF identification does not impose any additional power consumption on the transmitter side. Traditionally, RFFI is a three-stage procedure that involves extraction of the features, enrollment or storage of the features in a database, and the identification of the device based on the existing database. In the following sections, we describe different categories of RFFI methods. Since we mainly focus on LoRa RFFI, we limit our discussion to the main RFFI categories.

**Figure 6:**
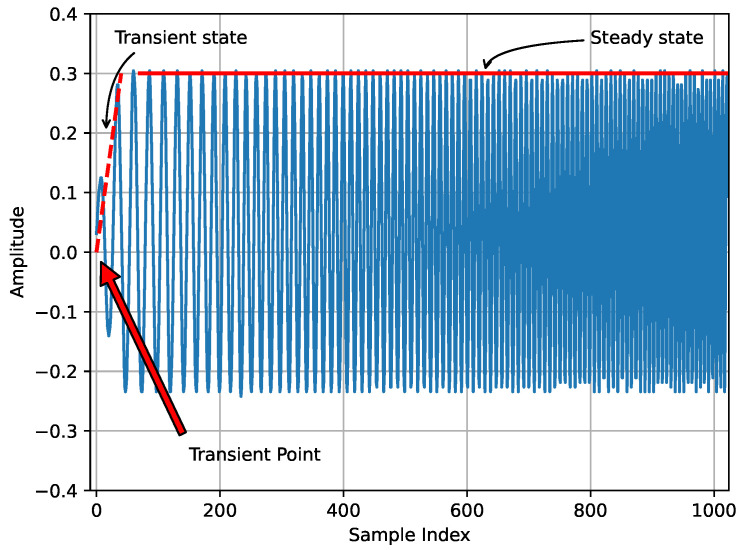
Transient and steady-states in a LoRa signal.

Transient-based RF fingerprinting techniques rely on capturing the transition period from the turn-on to the full amplitude of a transmitter signal, which occurs before the transmission of actual data in a signal [41]. The state between the transient point and the stable signal is called as transient state. Figure 6 illustrates the transient and steady states of a LoRa signal captured from a real Pycom LoPy4 device at 868.1 MHz, BW of 125 kHz, and SF 7. It is important to note that the plot is generated from the first 1024 samples of the LoRa IQ signal captured using Software-Defined Radios (SDR). The turn-on period of the signal is important as it can produce time-varying effects and transient distortions which result in features such as carrier frequency offset (CFO), and phase deviations. The transient of a signal can be extracted by performing the time-domain analysis of the captured signal and by setting an amplitude threshold.

On the other hand, the steady state means the period under which the signal remains stable. In other words, in steady-state RFF, the features are extracted from the modulated part of the signal. Figure 6, shows both the transient and steady-state parts of a signal. Extracting RF fingerprints from the radio signals is easier in steady-state RFF compared to the transient method. Additionally, transient RFF requires higher sampling rates and extremely precise detection of the starting point [18].

Modulation-based RFF refers to the radio signal metrics in the modulation domain such as modulation shape and spectral features that can be used as radio emitter identifiers. The advantage of modulation-based RF is that it provides a structured waveform with limited complexity, hence extraction of RF features is easier. For a detailed review of several RF fingerprinting methods, the reader is referred to [42].

Recently, deep learning-based RFFI approaches have been vastly studied in the literature [43,44,45]. DL-based RFFI requires the capture of real or synthetic IQ data from the emitters for training the algorithms. These algorithms have been found quite capable of learning the hidden hardware impairments from the RF signal and identifying a particular transmitter based on the training data. It has been observed that during deep learning model training, most of the time the steady-state portion of the RF signal is used as it provides stable RF features for the model to learn and recognize the emitter during the test. In this paper, we mainly focus on the deep learning-based RFFI for LoRa emitter identification.

### 3.2. RF Transmitter Hardware Impairments

**Figure 7:**
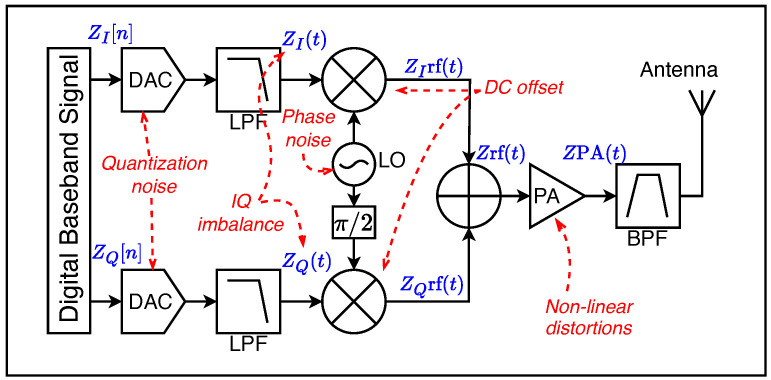
Direct conversion transmitter architecture (adapted from [47]).

Direct conversion transmitters have gained massive popularity in RF devices. Their typical architecture, shown in Figure 7, implements the parallel up-conversion of the I and Q components of the baseband signal by mixing them with two signals generated from the local oscillator at the same carrier frequency but shifted by 90°. Their main benefits are their simple design, low cost, and high-level integration [46]. These transmitters come with intrinsic hardware impairments such as frequency offsets, DC offsets, IQ imbalances, and power amplifier non-linearities as depicted in Figure 7. Such impairments are device-specific and can be used for the identification of a particular transmitter. Sankhi et al. [43] have utilized the RF impairments in WiFi signals to identify the transmitter using deep learning, whereas Zigbee-protocol-based device identification using hardware impairments and deep learning is proposed in [44].

The original LoRa patent [30] provides a block diagram with a direct conversion transmitter architecture and for this reason, their impairments have been used for LoRa device identification [47,48]. We discuss these impairments in this section.

#### 3.2.1. IQ Imbalance

IQ imbalance, also used interchangeably with IQ mismatch, is the deviation in amplitude and/or phase of the I and Q components of the signal. They are caused by imperfections in the Digital-to-Analog Converters (DAC), low-pass filters (LPF), and mixers, as denoted in Figure 7. These IQ imbalances are visible at the receiver and can be used as part of device fingerprints [47].

#### 3.2.2. Phase Noise

Phase noise is the result of imperfections due to local oscillators in an RF transmitter. Local oscillators are used to generate periodic signals, which are then utilized by mixers to upconvert the baseband signal to a passband signal. Ideally, the local response of the local oscillator should be a pure sinusoidal signal $cos(w_{c}t)$ with $w_{c}$ as the carrier frequency. However, oscillators are susceptible to temperature changes and operating conditions [49]. Hence, with changing environmental conditions, the oscillators are affected, which results in an imperfect response $\cos(w_{c}t+\eta \left(t\right))$, where η(t) is termed phase noise. For a detailed treatment of phase noise, one can refer to [38,47].

#### 3.2.3. DC Offset

There are two major sources of DC offset in RF transmitters. The first is the carrier leakage which comes from the local oscillator due to poor isolation between LO and mixer output ports. This leakage between the local oscillator (LO) and RF input path to the mixer is inevitable. This leakage signal, when mixed with the LO, generates an undesired DC component through self-mixing [50]. The second source of DC offset is the second-order non-linearity. These non-linearities originated due to the mixer and amplifier operation which can generate unwanted frequencies in the output signal.

#### 3.2.4. Carrier Frequency Offset

Carrier frequency offset (CFO) is also one of the RF impairments that have been used for RFFI [51]. CFO is the difference between the carrier frequencies of the transmitter and receiver caused by the oscillator of the transmitter. This shift in the carrier frequency causes a minor deviation in the received signal and can be used as an identifier for that particular device. Even after compensating the CFO, it has been observed that the accuracy reduces over time due to the hardware characteristics of the device [52]. Shen et al. [53] have used CFO along with time-frequency features for LoRa device identification using deep learning. The impact of CFO on RFFI system accuracy degraded over time.

#### 3.2.5. Sampling Frequency Offset

Sampling frequency offset (SFO) is the difference between the sampling rates of the transmitter and receiver that occurs during ADC and DAC conversion. Ideally, the sender and receiver sampling frequencies should be synchronized. However, in practice, there is a deviation that can result from hardware components and clock inaccuracy [54]. This causes the signal to be sampled at $n(T_{s}+\Delta T$) instead of the ideal case (nTs). Although, there has not been any study that explicitly exploited the SFO impairment for LoRa device identification it could potentially be used for this purpose.

#### 3.2.6. Non Linearity

Non-linearity in RF transmitters primarily arises from the power amplifier (PA) and mixer components. The PA plays a crucial role in amplifying the signal to an appropriate level for transmission. However, operating the PA in its linear region consumes excessive power, making the overall system less energy-efficient. As a result, the PA is often operated close to the saturation region, leading to non-linear distortions such as saturation and bandwidth expansion. These distortions are reflected in the instantaneous amplitude and phase output responses as the amplitude of the PA input signal varies [47].

### 3.3. LoRa-Specific Impairments

#### 3.3.1. LoRa Chirp Structure

LoRa is based on a chirp signal which varies linearly in frequency over time. The structure of this chirp signal can vary slightly due to the transmitter’s bandwidth, sampling rate, and hardware characteristics [55]. These slight variations from one transmitter to another can act as a device fingerprint.

#### 3.3.2. Unique LoRa Transceiver Design

**Figure 8:**
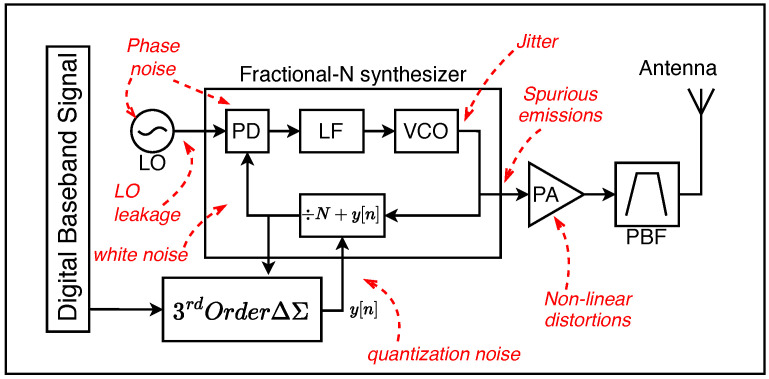
Possible transmitter architecture of Semtech’s SX127x (inspired from [56]).

According to Semtech’s LoRa transceiver datasheets [56], the architecture used in actual LoRa transmitters might not be a direct-conversion IQ modulator. The datasheet suggests that the LoRa modulation is performed directly in the control loop of a fractional-N third order ΔΣ Phase-Lock Loop (PLL), as shown in Figure 8. It is unclear whether future LoRa RFFI systems will take advantage of hardware limitations unique to this architecture. Digital PLLs are notorious for having jitters and frequency spikes; however, these impairments can be lessened by using larger loop orders, filtering, and extra canceling techniques.

#### 3.3.3. LoRa-Specific CFO Effect

Even though we already discussed the general impact of CFO on RFFI in Section 3.2.4, it has been observed that the CFO in LoRa transmitters can vary depending on the BW and SF parameters used. Semtech’s application note on optimal reference clock [57] reports on comparing three different quality local oscillators used in the end devices. It recommends for example that devices deployed in outdoor environments with higher exposure to temperature changes use high-quality oscillators to achieve a stable frequency offset range. On the opposite, to reach the lowest cost, some devices may be equipped with lesser-quality oscillators. Hence, the observed CFO can vary widely depending on the device application, an important consideration while dealing with RFFI.

#### 3.3.4. LoRa Symbol Timing Offset

LoRa is a propriety technology that does not make its inherent modulation or demodulation techniques. All the information about LoRa PHY that is available to use is a result of reverse engineering efforts. And it is found that one of the main issues in LoRa demodulation is accurate time and frequency synchronization. The unsynchronized timing reference can cause a symbol timing offset at the receiver which can be used as a fingerprint [58].

#### 3.3.5. RF Impairments under Low SNR

One of the benefits of LoRa CSS modulation is its performance under low SNR conditions. The dechirping process explained in Section 2.3.5 allows the extraction of the modulated data from signals below the noise floor. However, the noise is dominant in such scenarios, making it difficult for DL models to learn the RF impairments. It has been observed that the DL-based RFFI systems are severely affected by the low SNR LoRa RF data.

## 4. DL-Based LoRa Device Identification Using RFF

**Figure 9:**
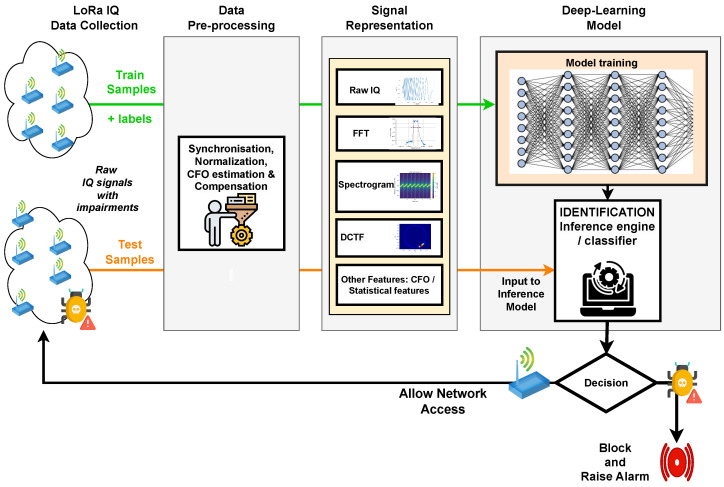
Block diagram of a typical deep learning-based LoRa radio-frequency fingerprinting identification architecture.

A DL-based LoRa RFFI system consists of various stages, i.e., LoRa signal collection, data pre-processing, model training and feature extraction, fingerprint storage and enrollment of new devices, and finally the identification of legitimate and malicious devices. Figure 9 shows a scheme of a DL-based LoRa RFFI system with various stages as mentioned. We describe these stages in detail below.

### 4.1. LoRa Signal Collection

The first step for the LoRa RFFI system is the capture of real LoRa signals. For experimentation purposes, this could be achieved by creating a test-bed scenario in an indoor or outdoor environment. In a real-world scenario, these signals can be captured from the LoRa devices already deployed in the network. This can be achieved using equipment capable of capturing and processing the received signal. Normally, USRP SDR from Ettus Research [59] are used as LoRa receivers. These SDRs can be configured using the Matlab platform [60], or by the GNU radio companion tool [61], which is open-source and more user friendly. The LoRa signals are captured and saved as a complex IQ format for further processing.

### 4.2. Data Pre-Processing

Since DL-based systems are usually not good at learning from the raw data collected from different devices, a crucial step consists of pre-processing the raw data to make DL model life easier. For LoRa IQ signals, the pre-processing step may involve synchronization, CFO estimation and compensation, and normalization and data partitioning. These pre-processing steps must be applied to training and test sets. We briefly describe those steps below.

#### 4.2.1. Frame Synchronisation

Accurate synchronization of the signal is critical for locating the correct starting point of the preamble and ignoring the noise channel. Different LoRa synchronization methods are used in open-source LoRa PHY [31,32,62]. The Schmidl–Cox [63] synchronization method is implemented by [64] while Robyns et al. [32] identify the starting point by cross-correlating the expected and measured instantaneous frequencies of the signal.

#### 4.2.2. CFO Estimation and Compensation

Shen et al. [64] implement CFO estimation methods in their work to show the impact of CFO on the performance of DL models. Their coarse CFO estimation is just the mean instantaneous frequency of the received preamble, under the assumption that LoRa relies on up-chirps with linearly increasing frequency. The authors believe that CFO compensation is necessary as it varies over time and can affect the performance of the DL model.

#### 4.2.3. Normalization

The input samples are not always in the same range or scale due to different transmission ranges and the action of the receiver’s automatic gain control (AGC). This can create bias during model training. Therefore, normalizing the data samples to bring them on the same scale is necessary. Root mean square (RMS) normalization is commonly used for RF signals [53,65].

### 4.3. Signal Representation

Providing raw IQ samples as input to the DL model is computationally expensive. It can also affect accuracy, as time-domain features depending on the environment might be learned by the model. Extracting frequency-domain representations or spectrograms from the IQ samples has been shown to improve the performance of DL-based LoRa RFFI systems [65,66]. Therefore, deciding the LoRa signal representation for the DL model is also a major stage.

#### 4.3.1. Time-Domain

**Figure 10:**
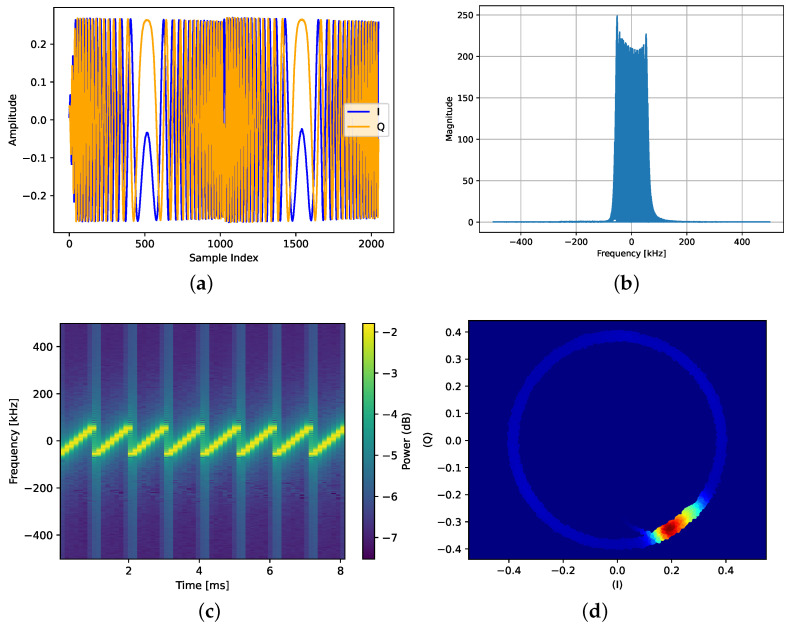
Four different representations of the LoRa IQ signal. (**a**) Time-domain (IQ); (**b**) Frequency-domain (FFT); (**c**) Time-frequency domain (spectrogram); (**d**) Differential Constellation Trace Figure.

LoRa signals captured in real time are in raw in-phase and quadrature (IQ) samples. I(m) and Q(m) are the real and imaginary parts of the received signal that carry its amplitude and phase information. These components are extremely useful in the digital signal processing field. Most authors use raw IQ samples as input to deep learning models for radio frequency fingerprinting identification [47,48,67]. Figure 10a plots, in the time-domain, the two first preamble symbols of a LoRa signal sent with SF 7, BW 125 kHz, and captured at a sampling rate of 1 MS/s.

Amplitude and Phase (A/ϕ) is another time-domain representation. It has been used by [47,68] as an input signal to the DL models. However, this representation has not proven to be the best for LoRa device identification using DL.

#### 4.3.2. Frequency Domain (FFT)

Deep learning models are not able to learn the frequency characteristics of signals when they are provided in a time-domain representation. Therefore, converting a time-domain signal into a frequency-domain using the Discrete Fourier Transform (DFT) allows exposing the important frequency features to the deep learning models and as a consequence helps to improve identification performance. The Fast Fourier Transform (FFT) which is an efficient implementation of the DFT outputs the magnitude and phase of each frequency component in the signal. Figure 10b shows the frequency-domain representation of a LoRa signal. The frequency coefficients resulting from applying the FFT on LoRa IQ signals have been used to train DL models for RFFI purposes [47,65].

#### 4.3.3. Time-Frequency Domain (Spectrogram)

The spectrogram is used to reveal the time-varying characteristics of the signal spectrum as a 2D image. Deep learning models such as CNNs perform exceptionally well on images. Hence, spectrograms have proven to be an efficient and excellent choice as input to deep learning-based RFFI systems [53,64,65]. Therefore, generating a spectrogram from the LoRa signal can be a better choice as an input to such models. The spectrogram is generated using Short Time Fourier Transform (STFT), where a small segment of the signal in time is taken and DFT is applied. The DFT is applied on all the signal segments shifted in the time window. Then, the power spectrum of each component is computed. Figure 10c shows the spectrogram of the real LoRa signal similar to the one used for plotting time-domain representation in Figure 10a. We used a window length (N) of 256 with an overlap (R) of 128. The length of the window function, overlapping size, and shape of the window function are adjustable parameters and affect the overall size of the spectrogram. A longer window produces a narrowband spectrogram and results in less precision in time. On the other hand, a shorter window produces a wideband spectrogram but takes fewer DFT points resulting in poor frequency resolution. These parameters affect the necessary feature extraction and can have an impact on the DL models’ performance.

#### 4.3.4. Differential Constellation Trace Figures (DCTF)

Another 2D signal representation that has proven useful in RFFI is the Differential Constellation Trace Figure [69]. The core of this approach is to apply a differential operation on the signal, where the samples are multiplied by a delayed version of themselves before being plotted in a polar form like in a constellation diagram. This results in a circular-looking figure that highlights variations in the signal caused by imperfections in the transmitter, such as I/Q imbalance, non-linear behaviors, and carrier frequency offsets. The differential operation causes the signal to fold over itself, resulting in an area of high density whose shape is a fingerprint of the transmitter. The benefits of the DCTF are that it requires no frequency and time synchronization. Moreover, it has proven effective with simple clustering methods and 2D deep learning models. A DCTF plot of a LoRa IQ frame captured at SF 7, BW 125 kHz and sampled at 2 MS/s is plotted in Figure 10d.

### 4.4. DL Model Training

Deep learning has achieved massive success in terms of applications in every aspect of life. Especially, with the invention of powerful computing machines such as graphical processing units (GPU), the training and deployment of deep neural networks have become easier and faster. Deep learning has now rapidly been adapted in wireless network applications because of its proven state-of-the-art performance in natural language processing (NLP) [70], speech processing, and computer vision [71].

Once the data have been pre-processed, the next step is to choose the DL-based model that provides the best performance. DL algorithms have proven to be excellent at extracting features from the data without needing manual feature engineering. CNN is by far the most commonly utilized DL algorithm for LoRa device identification. In addition to CNN, other DL algorithms such as LSTM, ResNet, and Transformers have also been used for RFFI purposes. However, various factors such as efficiency, training complexity and cost, time computation, and scalability affect the choice of DL models.

### 4.5. Device Identification

This is the crucial stage in a DL-based LoRa RFFI system. The DL model, which is trained, can act as an inference and decide on the new test samples whether they are already in the database or not. If the device label has not been identified correctly, the algorithm will reject the authentication and raise an alarm. Only legitimate devices that were already present during the training will be allowed access to the LoRa network and be authenticated using the existing cryptographic authentication.

## 5. Critical Literature Analysis

**Figure 11:**
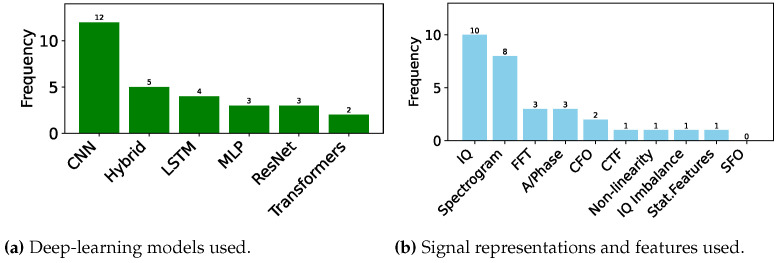
Deep learning models, signal representations, and features.

In this section, we describe all the studies published in the domain of DL-based RFFI for LoRa devices. We divide this section into subsections according to the DL model being used for classification. In addition, a summary of the models used in the literature is presented in Table 1, where we list down the models concerning the year of publication from the oldest to the most recent work. We also show the frequency of each model and the signal representation being used in Figure 11a,b, respectively. We further elaborate the published literature in Table 2 by their approach, signal representation, and features used as input to the model. We also put our remarks on the findings and gaps in the studies.

### 5.1. CNN-Based LoRa RFFI

*Convolutional neural networks (CNNs)* [86] are excellent at extracting features from image data and have been widely used for computer vision tasks. A CNN consists of multiple layers such as the convolution layer, pooling layer, activation, flatten layer, and classification, also called the fully connected (FC) layers. CNNs have been used for RFFI in multiple works [47,65,77]. In this section, we discuss all the studies that focused on using CNN for LoRa device identification using RF fingerprints.

A detailed and comprehensive study of deep learning-based LoRa device identification using radio frequency fingerprinting is provided in [47]. A massive data collection is performed using 25 Pycom devices with Semtech SX1276 LoRa transceivers, under five different scenarios, including different days, environments, configurations, distances, locations, and receivers. The receivers include two USRP B210 SDRs. The details of the dataset are discussed in Section 6. Specifically, for the first time, out-of-band distortion caused by LoRa devices has been proposed for device identification using DL. Out-of-band distortions refer to the unusual behavior caused by the hardware impairments (see Section 3.2) in the signal beyond the spectral width. Using different signal representations such as IQ, FFT samples and (A/ϕ) components of the LoRa signals, a CNN model was implemented for classification. Using same-day data for training and testing, the model achieved an accuracy of 84% using FFT signals, compared to 78% with IQ samples and 60% with (A/ϕ). However, when testing the model’s generalization ability to test data from a different day, the performance is severely degraded, irrespective of the signal representation. Moreover, the impact of the indoor and outdoor environment is also analyzed, and it is concluded that with an accuracy of 80%, FFT outperforms IQ and (A/ϕ) representations. However, whatever the signal representation, the proposed CNN model suffers severely from changes in the environment, location, and parameters.

Qi et al. [77] exploited these RF impairments in real LoRa IQ signals and used them to identify the emitter using a CNN model. The amplitude and phase imbalance along with statistical features (mean, variance, standard deviation, skewness, and kurtosis) are used as input to train the CNN. The data were collected from eight LoRa transmitters using USRP-2942R. A CNN model with one convolutional layer and two fully connected layers is used for the identification. To train the model, an SGD learner with a learning rate of 0.01 was chosen with epochs up to 300. On average, the model achieves an accuracy of 93.25% on the test set.

Shen et al. [53] calculated spectrograms from the LoRa signals and employed them as inputs for a CNN model. In addition, the impact of CFO drift on the model performance was analyzed as a function of the environment and the days. It was found that CFO drift can cause system stability issues and degrade the classifier’s performance. Therefore, CFO compensation is an essential technique for an RFFI system. A CNN-based classifier is proposed to perform classification tasks based on CFO. At the initial stage, a reference CFO database is created. For all the devices under classification, their CFOs are estimated and compared with the database. When the difference between the estimated CFO of a device and the corresponding reference CFO stored in the database is large, the probability is set to zero, and vice versa. The device with the highest probability is selected as the final predicted label. The CFO varies over the days and this drift in CFO causes misclassification. Using the same day’s data for the training and test models gives an accuracy of 99.57%, whilst using the first day’s data for training and the second day’s data for testing gives an accuracy of 78.84%. It shows that the proposed model was not able to generalize well to data collected on different days.

Zhang et al. [74] provided a comprehensive discussion on the modeling of PA non-linearities and IQ gain imbalances for RFFI systems. Based on these impairment models, simulations were conducted for experimental validation. However, frequency drifts caused by the oscillator were not considered stable features for DL-based RFFI systems. This was concluded after hardware experimentation over three months. Therefore, only PA non-linearities and imbalances in the I and Q branches of mixers were used to train a CNN model. The proposed CNN model consists of five convolutional layers followed by one flattened and two FC layers. Based on the mentioned impairments, the proposed CNN model achieved up to 99% (resp. 89.3%) accuracy on 50 (resp. 200) devices. It was also observed that low SNR affects the accuracy of the model. Moreover, the accuracy degraded largely down to 20% when the model was tested on data from different receivers.

Gao et al. [80] proposed a CNN-based LoRa transceiver identification system using spectrogram and CFO. Unlike [64], the authors in this paper merge CFO with spectrogram pixels by converting the spectrogram matrix into factors using the singular value decomposition method. It is a technique used for the factorization of real and complex matrices. For data collection, 10 MDots mbedded LoRa nodes [87] with SX1272 transceivers were used as transmitters. The BladeRF 2.0 SDR was used as the receiver, operating at a frequency of 915 MHz and a sampling rate of 2 MS/s. The transmitters were configured to send with SF10 and 500 KHz BW. Spectrogram along with the CFO is provided as input to a six-layer 1D-CNN model with batch normalization and ReLU activation. The learning rate for an Adam optimizer was set as 0.0003 and the batch size was fixed at 32. The results show that the proposed system achieves better performances than the existing work using spectrograms [65]. In the worst-case scenario, the proposed technique achieves 13% higher accuracy when training and testing are performed in different locations and on different days. Compared to [65], which provides 67.53% accuracy, the proposed system reaches 80.77%.

In [82], the authors tackle the issue of needing to retrain the model from scratch for new devices. For this purpose, they introduce two techniques for transfer learning-aided DL-based LoRa RFFI system. In one of the techniques, a pre-trained CNN model is used on the data collected from devices already in the LoRa network and then tested on the signals from the new devices. But the model is fine-tuned with 15 signals from unknown devices and after this fine-tuning process, the classifier can identify the new LoRa devices with over 90% accuracy. In the second method, simple deep metric learning is used where the enrollment data are collected from the legacy IoT devices, and then the extractor is attached to the k-NN classifier. The proposed methods are also tested on data collected from different locations. It is concluded that distance metric learning is an efficient method as it requires less computational resources compared to fine-tuning transfer learning methods. The methods were implemented on LoRa data collected from 40 devices and the data were augmented to increase the samples.

Gaskin et al. [75] proposed a CNN-based hybrid neural network architecture called Tweak which can perform well when tested on different domain data. Hence, it is said to be a domain-agnostic DL model for RFFI. This is achieved using a small number of data samples from the target domain for training. A deep metric learning and calibration process is implemented to enable model learning from different domains. Considering the real-time scenario this significantly affects the data requirements and training time consumption. The test data were from 15 other devices and the model was evaluated using TPR and FPR. As expected, the proposed model was able to achieve high TPR when trained and tested under the same conditions, i.e., same day, transmitter, and receiver. On the other hand, the performance degraded under different day scenarios.

In [81], a two-layer CNN classifier is evaluated using LoRa IQ data from eight devices. The data were captured using HackRF SDR under wired and wireless scenarios. The presented CNN model achieved high accuracy up to 99% in the wired scenarios, whereas in the wireless scenario, the model’s performance was reduced to 97%. The authors also evaluated the model by introducing a synthetic IQ imbalance in the data. It is shown that the CNN model was able to achieve 99.90% accuracy on the data impaired with IQ imbalance. However, one of the major drawbacks of the study is the amount of data used for training and testing the model. The CNN model was trained using only 600 preambles per device.

Guo et al. [84] proposed a novel RF fingerprinting technique using cross-power spectral density (CPSD) based on the cyclic shift property of LoRa chirps. The CPSD fingerprints are used to train a 1D-CNN model which achieves notable accuracy of 98.42% in NLOS scenario while 98.35% in LOS. The data were collected from 60 LoRa transmitters using USRP B205 SDR over a long period of 3 years. Overall, 35,870 frames were captured from all devices. The number of frames per device captured is not clearly mentioned.

In [83], the variational mode decomposition (VMD) technique is used to decompose the LoRa signal into components each representing a unique signal characteristic. The VMD is generally useful for non-stationary signals such as LoRa to analyze each local component’s signal frequency and amplitude variations. However, in this paper, the authors focused more on the residuals of the VMD and trained the CNN model. The advantage of the proposed method is the reduced dimensionality of the spectrogram image that is being used to train the CNN model. The reduced size results in lower computational overhead and faster training of the DL. The authors used the RF data from only 10 devices from publicly available datasets [64]. The implemented CNN model achieved an accuracy of 91.90% using the residuals while it reached 90.70% while trained with the original spectrogram image generated using a window length of 256. However, the VMD technique is generally very slow if the data have long sequences. Hence, if trained on 100s of devices, the computation time will increase.

From these studies, we can conclude that the CNN can perform excellently on LoRa IQ signals and spectrograms and in some cases achieve high accuracy up to 99%. However, it is sensitive to the variations in the environment, LoRa configuration, and time of the signal capture.

### 5.2. ResNet Based LoRa RFFI

*Residual Network* ResNet [88] is CNN-variant. ResNet was introduced to solve the vanishing gradient issue with deeper networks. The ResNet consists of residual blocks, each with some number of convolutional layers. The key property of the ResNet architecture is the introduction of skip connections which enable the training of deep neural networks with hundreds of layers. The idea of the skip connection is to bypass the information from certain layers and transfer it directly from one layer to another. Simply put, the output of the previous layer is bypassed and added to the output of the current layer.

The authors in [64] considered the open-set problem and presented a channel robust deep learning model for LoRa device identification using RFF. The proposed RFF extractor consists of three stages namely, enrollment, rogue device detection, and identification. Deep metric learning is exploited to train the RFF extractor which provides excellent generalization ability on previously unseen devices. The RFF extractor is trained only once and can be used to enroll and identify the devices repeatedly. The RFF database is maintained to allow devices to join and leave. To tackle the wireless channel distortions, data augmentation, and channel-independent spectrogram approaches are implemented. The extractors were able to identify the device from the same-day data with an average accuracy of 95%. Whilst in different day scenarios, the accuracy drops to 75.80%, to say the least. In a similar case study [66] by the same authors, the closed-set problem is also included.

In [85], a low complexity optimized version of the ResNet model proposed in [64] is presented. It is shown that the existing model can be optimized into a lightweight model with fewer parameters without compromising much on the performance. The optimized model has 75% fewer parameters than the original and can still provide an accuracy above 97% over 30 devices. In addition, a comparative analysis of different signal representations using a 1D-CNN model is also provided. Among the IQ, FFT, and A/ϕ, the IQ has achieved the highest accuracy for the 1D-CNN model.

An ensemble learning model based on Resnet34, Inceptionv3, and Densenet121 is used in [79]. The LoRa signals were converted into a spectrogram using the STFT with a Kaiser window size (*N*) of 128 and α factor of 4. The proposed hybrid model achieved an accuracy of 95.1% on the test set outperforming the individual models mentioned above with an accuracy of 91.6%, 93.2%, and 93.3% respectively.

Zhang et al. [76] proposed a hydride model called deep fractional scattering network (DFSNet) to extract radio frequency fingerprints (RFF) features hidden in non-stationary LoRa chirp signals. The authors used an open-source dataset provided by [47]. However, the authors only extract 6000 frames per device and convert the IQ samples into spectrograms using STFT. The proposed hybrid model was compared with ResNet-1D, 2D-CNN, and AlexNet-1D models as a baseline. It can be observed that the proposed DFSNet model outperforms other models and achieves a 17.47% increase the model achieves an overall accuracy of 98.5% compared to 96.6%, 96.8%, and 95.3% by ResNet-1D, AlexNet-1D, and 2D-CNN, respectively.

### 5.3. MLP-Based LoRa RFFI

*Multilayer Perceptron (MLP)* is a very popular feed-forward neural network architecture that can be used to approximate any continuous function [89]. MLP has been effective in applications such as fraud detection [90], network intrusion detection [91], and medical diagnosis and treatment recommendation [92]. MLP has been used for LoRa RFFI in [65,67]. However, MLP is a simplistic neural network that does not deal well with complex data structures such as 2D images.

A zero-shot learning-based methodology for LoRa device identification using RF fingerprint is presented by Robyns et al. [67]. Zero-shot learning is a machine learning technique where the model is tested on the classes with zero samples in the training dataset. The entire LoRa signal is used in the study compared to various other studies which utilize only the preamble part of the signal. A payload of 4 bytes with content not mentioned was used to transmit over the LoRa signal. However, to avoid any bias due to the payload, the payload bytes were randomized. In total 22 LoRa devices from different manufacturers were used for creating the testbed scenario. The data were collected during 30 days. CNN, MLP, and SVM classifiers were implemented using the collected data. Each of these models was trained up to 10,000 epochs. The proposed zero-shot learning technique achieves an accuracy ranging from 59% to 99% per symbol using identical chipsets. The effect of sample rate, time, and distance are studied. The performance of the models was reduced when tested with data from a different transceiver. The impact of the distance is also negative on the proposed classifier in this work. One of the major issues with this research is that the authors do not consider the effect of CFO.

In [65], MLP, CNN, and Hybrid models are evaluated using real-time LoRa signals. The raw IQ signals are converted into FFT and spectrograms to be provided as input to these models. The data were pre-processed using synchronization, normalization, and CFO compensation. CFO estimation and compensation are shown as inevitable components of LoRa device identification using RFF. During training a reference CFO database is created and then compared with the estimated CFO of each device during the test phase. There are two hypotheses considered in this study: (1) if the estimated CFO is less than the reference threshold, the probability of that particular class is set to zero, since the difference between the reference and estimated CFO of a particular device is large (2) If the estimated CFO value of the incoming signal is equal to or greater than the reference CFO, the probability of that particular device is high and the label is maintained. This calibration allows the model to select the label with the highest probability. It can be observed that CFO varies over the days and this variation affects the performance of deep learning models. Overall, the hybrid-CNN model outperformed other models tested by the authors and can achieve 98.11% accuracy when tested on IQ samples.

Jiang et al. [69] compared MLP, CNN, and SVM with a clustering-based LoRa device identification using constellation trace figure features. The proposed method relies mainly on the features from differential constellation trace figures with a pixel density higher than the defined threshold. The implemented models were also compared on different sampling rates of the LoRa IQ signals. The proposed clustering-based method outperformed the MLP, CNN, and SVM baseline with an accuracy of 99.67% at 10 MS/s. While MLP achieved 96.3%, and CNN reached 94.80%. One of the drawbacks of the study is that the number of LoRa devices was limited to only six.

### 5.4. LSTM Based LoRa RFFI

*Long Short-Term Memory* (LSTM) was introduced to address the critical issue of vanishing gradients in simple RNNs when trained on longer time steps. The structure of an LSTM cell is based on gates [93]. One of the advantages of LSTM is that it can hold information in memory for the long term. This is possible with the memory blocks attribute of an LSTM, which controls the inflow and outflow of the information using gates. The output of the LSTM network is dependent on long-term memory (cell state), output from the previous time-step (hidden state), and input at the current time-step [94]. LSTM has been used for radio frequency fingerprinting identification [95] and modulation recognition [96].

LSTM-based IoT device classification is proposed by Das et al. [72]. LSTM architectures with varying depths were evaluated for this task. It is found that the LSTM with two layers provides the best accuracy results of up to 99.58% followed by one-layer LSTM (97.45%) and three-layer LSTM model (96.60%). It is further concluded that increasing the number of LSTM layers causes over-fitting of the model. LSTM unit length is an adjustable parameter that actually reflects the number of cells or memory blocks in an LSTM layer. Choosing a larger unit length can capture intricate patterns from the signal data but also increases the complexity of the model. In this study, the authors chose 2048 as an optimum LSTM unit length. A test bed was created at the university campus building using 29 Semtech SX1276 devices as LoRa transmitters and one SX1257 as a receiver. All LoRa transmitters are considered as low power adversary nodes. In addition, to emulate the attacks from high-power adversary nodes USRP N210 radios [59] were also used for data collection. Furthermore, the evaluation encompassed both the Line-of-Sight (LOS) and Non-Line-of-Sight (NLoS) scenarios. To evaluate the robustness, the model was evaluated using noise levels between −5 to 10 dB. At −5dB, the model was able to identify the device with an accuracy of 88%.

In [68], a data augmentation method named *DeepLoRa* is introduced to improve the robustness of the device authentication method using RF fingerprinting. The collected data are represented as IQ, (A/ϕ), and spectrogram and provided as input to CNN and LSTM models. The proposed augmentation technique is based on the ITU channel model for outdoor to indoor and vehicular environments specified in ITU-R recommendation M.1225 [97]. The data augmentation framework introduced in this work provides flexibility to choose multiple wireless channels by tuning the parameters such as delay spread, SNR values, multipath fading type, and channel environment. The raw data are first collected from 100 LoRa devices and then passed through the DeepLoRa augmentation framework to increase the robustness of the model while training.

In addition, three LSTM models with different levels of complexity, one 1D CNN and one 2D CNN model were implemented in this study. The models were trained and tested using both the preamble and the payload part separately. Furthermore, the models were tested on different day scenarios and it is observed that failed to achieve commendable results using only payload, especially on different day scenarios. With only 10 devices, the best-performing LSTM model achieved only 16% accuracy while with 100 devices it dropped to 3%. On the other side, 1D-CNN achieved 28% accuracy with 10 devices and dropped to 5% with 100 devices. The results with the preamble data degraded more than the payload part. Multiple experiments were conducted on the augmentation of the data and models were trained using those data scenarios. Overall, DeepLoRa seems to have improved the accuracy of the models from only 19% to 36% on a different day scenario. This can be contrasted with the same-day scenario where the accuracy reaches up to 91% with 100 devices.

### 5.5. Transformer-Based LoRa RFFI

*Transformers* [98] are sequence-to-sequence architectures that are proving to be extremely efficient for the tasks of language translation and text generation. The transformer model consists of an encoder and decoder, both stacked with multiple layers. Transformers are different from Recurrent Neural Networks (RNNs) in that they do not rely on LSTM or Gater Recurrent Unit (GRU) for performance and use attention mechanisms, which perform much better than the mentioned RNNs. The readers can refer to [99] for a detailed explanation of attention-based models. Transformers have been utilized for radio frequency fingerprinting in [73,75] which are discussed in the subsequent paragraphs.

The LoRa Adaptive Data Rate (ADR) mechanism results in varying preamble length packets whereas deep learning models such as CNN cannot scale to classify varying-length packets. Transformers are capable of dealing with sequential data of varying lengths. In [73], a transformer model is proposed for the LoRa device identification using LoRa signal captured with different SF configurations. The IQ samples were collected from 10 LoRa LoPy and Dragino LoRa shields all consisting of Semtech SX1276 chipsets. Each device is configured at 125 kHz bandwidth, 868.1 MHz center frequency, and a 0.5 s transmission interval. The data were captured device by a device with SF values of 7, 8, and 9, respectively, where each device transmitted 3000 frames per SF. The captured LoRa data were augmented using online and offline methods. In an online augmentation technique, the data are augmented in mini-batches during the training whereas offline augmentation involves passing the data through the AWGN channel before training.

The data were pre-processed and noise was introduced using AWGN range between 0 and 40 dB. The transformer is trained on augmented data with various strategies. A multi-packet inference approach is also discussed to evaluate the classifier on low SNR values. Multi-packet inference is a method where multiple LoRa transmissions from a device are considered for the prediction. The probabilities of the multiple packets are averaged together to derive the label index with the highest index.

It is found that the transformer provides better results on all SF configurations using SNR values over 30 dB. Also, augmenting the data provides better results with lower SNR values compared to the data without data augmentation. The proposed system provides nearly perfect accuracy at SNR over 30 dB, whereas the accuracy reduces to 60% but with multi-packet inference, it reaches up to 90% 10 dB. It is found that multi-packet improves classification accuracy but using more than 10 packets shows no improvement.

Shen et al. [78] elaborated on an RFFI system that can process signals of variable lengths and leverage data augmentation to train noise-robust models. A flatten-free CNN, LSTM, GRU, and transformer models were chosen for this study. The authors collected a large number of LoRa packets in the training stage to generate a training dataset. They collected LoRa packets of various spreading factors to ensure that the system can handle different SFs in the inference stage. Specifically, they used SFs 7, 8, and 9 as examples. The collected packets were pre-processed and used to train the proposed neural network architectures. The results showed that the proposed system can achieve high classification accuracy in low SNR scenarios. Specifically, it is observed that the online data augmentation strategy has a positive impact on the performance of the model and can significantly improve the model’s robustness to noise. In addition, the multi-packet inference approach can further increase the accuracy by over 20%. The results also showed that the classification accuracy decreases as the SNR decreases, which is expected as the signal becomes more distorted by noise.

### 5.6. Lessons Learned

The most striking lesson learned from the literature survey is that DL-based LoRa RFFI systems remain quite sensitive to changes in the environment and the parameters. All models implemented in the state of the art have suffered severe performance degradation when they are trained and tested with data collected in different environments. Some of the existing studies propose partial solutions to this challenge, but further work is required in this domain to make RFFI systems more robust.

It can be observed from Figure 11a that most of the time, CNN has been the first pick of the researchers for the LoRa device identification, whereas LSTM, ResNet, MLP, and Transformers have also been used. In addition, hybrid structures using models such as CNN and ResNet have also been implemented.

In Table 3, we provide an in-depth analysis of different DL model architectures employed for the LoRa RFFI system. We dive deeper into model depth, the activation function chosen, and the optimizer algorithm used. We also point out if the code has been released for reproducibility. Additionally, it is noted that the majority of articles do not fully explain every parameter used in the DL model, including data distribution, time batch size, and training epochs.

We also analyze the different models used, the signal representation chosen to train the models, and their accuracy performance in Table 4. Since different models and scenarios were used in the articles; hence, it was difficult to show the performance under all scenarios. We only report the best accuracy results presented in the paper. It can be observed that most models achieve higher accuracy results up to 99% in certain scenarios. The best results are achieved when the train and test data have been used from the same scenario, i.e., (same day, same environment, and same parameters).

In addition, selecting the right signal representation (Section 4.3) is critical. While IQ representation has been widely used, as can be seen in Figure 11b, the spectrogram, which essentially shows the time-frequency characteristics of the LoRa modulation, appears to be the best representation in terms of accuracy, provided the right DL model is chosen. In addition, FFT and A/Phase have also been chosen as signal input for deep learning models. In some of the cases, impairments such as CFO, non-linearity, and IQ imbalance have been used as separate features for training deep learning models. One of the authors also used fractional wavelets as input to train the deep learning model.

CFO has been envisioned as a key device-specific impairment that can be used to identify the emitter using DL. However, the literature does not explicitly support the use of CFO as an independent feature for training DL since it is time-variant and therefore is not a stable feature. In addition to this, CFO estimation and compensation are presented as mandatory pre-processing steps. Similarly, statistical features of the captured signal have been used along with hardware impairments to help improve the RFFI performance. However, these statistical features might not be stable with time and hence might prevent the DL model to generalized.

Most of the existing papers in the literature are focused on closed-set problems. In a close-set RFFI problem, for any rogue devices to join the network, the model needs to have prior information about them. Since the model is trained with such devices, it will allow the device to join the network based on similar features to the already included devices in the database. Every time a new legitimate device must join the network, the DL model needs to be retrained to learn its features. This is time-consuming and impractical for a real-world implementation. On the other hand, we refer to the open-set problem when an RFFI system refers to the identification and enrollment of devices with no prior information. There has been very little work on the open-set problem to date.

Our literature survey has exhibited several quite diverse approaches. Even though some of the DL models used are shared by some authors, each one makes use of its specific set of features (CFO, IQ imbalance, DC offset), signal representations (IQ, FFT, Spectrogram, wavelets), pre-processing steps (CFO compensation) and deep learning techniques (data augmentation, deep metric learning, transfer learning, ensemble learning). Comparing the achieved performance of these studies is therefore difficult without their application to a common/standard database.

## 6. LoRa RFFI Datasets

Data availability is one of the main issues for LoRa RFFI research. Only a few datasets collected from real LoRa devices are available publicly. In this section, we discuss the LoRa RFFI datasets in detail and summarize them in Table 5. For each dataset, we describe the test-bed scenario including the number and type of the devices used, the LoRa parameters, the environment, and whether or not the dataset is publicly available. We also found out that all datasets at least provide the captured signal as IQ samples. The datasets described here are organized according to the year of publication. Certain datasets are repeatedly utilized by researchers in their work, for instance, the dataset introduced in reference [64].

### 6.1. Dataset I

Dataset I discussed in [67] was collected using 22 LoRa transceivers and Ettus Research B210 USRP SDR. The LoRa transceivers were configured at an operating frequency of 868.1 MHz, a spreading factor of 7, and a code rate of 4/8. Each of the transmitters was set to transmit a Lora frame with a payload size of 4 bytes resulting in 36 symbols per frame. To eliminate any DC bias effect of USRP components, it was tuned at 868 MHz instead of 868.1 MHz. The data were collected on different days within 30 days to observe any effects due to variation in the channel. In addition, for the sake of analyzing its effect, different sampling rates were used across the collected datasets: 1 MS/s, 2 MS/s, 5 MS/s and 10 MS/s. A total of 1,798,803 LoRa symbols were collected on different days. The difference between most of the existing datasets and this dataset is that they collected the whole frame instead of only the preamble part of the LoRa frame. This dataset, however, is publicly accessible and can be accessed from Zenodo (https://zenodo.org/records/583965, accessed on 25 May 2024).

### 6.2. Dataset II

In [69], the authors collected Dataset II using a total of six Semtech SX1278 LoRa transmitting modules and a USRP SDR as receiver operating at 433 MHz. The LoRa devices transmitted the signals at an SF value of 7, BW of 125 kHz, and the USRP was configured at a sampling frequency of 5 MS/s with a frame interval of 50 ms. There are no further details of the dataset provided in the paper and the dataset is not publicly available.

### 6.3. Dataset III

Dataset III was collected at Oregon State University by the authors of [47]. For this purpose, they had set up a test bed consisting of 25 identical Pycom IoT devices used as transmitters and one single USRP B210 SDR as a receiver. The LoRa transmissions were performed at a center frequency of 915 MHz using a single spreading factor of 7, BW of 125 kHz, and a coding rate of 4/5. The SDR was driven by the GNU radio software to sample the LoRa transmissions at 1 MS/s. The authors considered seven scenarios to measure the impact of environmental conditions on the performance of deep learning models. The data were collected both indoors and outdoors, on different days, and under different device configurations. In addition, the location of the devices was also changed and a different receiver was used for one scenario.

The dataset consists of both the IQ time-domain signals and their corresponding FFT representation. The collected data are provided as separate binary files using the SigMF format (https://github.com/sigmf/SigMF/blob/sigmf-v1.x/sigmf-spec.md, accessed on 25 May 2024). Each file is accompanied by a plain-text JSON meta file which provides the recording details such as sampling rate, time and day of recording, and duration. The complete dataset consists of 16,300 files occupying around 1.2 TB of storage. Each of the transmissions for all scenarios is saved as a separate file and then another file is created for the FFT of each transmission. The dataset is publicly available and can be accessed at Oregon State University Cloud (https://research.engr.oregonstate.edu/hamdaoui/RFFP-dataset/, accessed on 25 May 2024).

### 6.4. Dataset IV

Dataset IV [65] is collected using 25 LoRa transmitters from different models including five SX1272MB2xAS Mbed shield, five SX1261 MB2xAS mbed shield, five Pycom FiPy, five Pycom LoPy, and five Dragino SX1276 shields. The transmitters were configured at 868.1 MHz, SF7, BW 125 kHz and a code rate of 4/8. On the receiving end, USRP N210 SDR was used with a sampling rate of 1 MS/s. The data collection process was continued over a period of several months from April to November. This dataset is created using a wired scenario where the LoRa device was connected with USRP SDR using a 40 dB attenuator. The dataset is not publicly accessible.

### 6.5. Dataset V

Dataset V [74] is collected using 5 SX1272MB2xAS LoRa shields connected to a USRP N210 SDR by a 40 dB attenuator. The purpose of the dataset was to analyze the effect of CFO variations due to the change in oscillator operations. For classification purposes, a separate synthetic dataset was created using different channel environments. The dataset was created for 200 devices with varying SNR values. However, the paper does not provide details about the other LoRa parameters considered during the simulation. The dataset is also not publicly available.

### 6.6. Dataset VI

Dataset VI [68] is a massive dataset of 1TB collected using 100 LoRa devices. Pysense sensors were connected using 100 LoRa-enabled FiPy radio transceivers. A USRP N210 SDR with a CBX 1200–6000 MHz daughterboard is used as the receiver and the carrier frequency was set at 902.3 MHz. The data were collected over multiple days in different environments. The LoRa preamble and payload were extracted to analyze the effect of both individually on RFF. Each of the LoRa transmitters sends 100 consecutive packets separated by 10 ms in a single burst. An indoor environment test bed is created in the office whereas the outdoor dataset was created outside the building. The transmitters were placed outside of the building while the receivers were inside. The dataset is publicly available and can be accessed at InterDigital (Source: https://www.interdigital.com/data_sets/lora-radio-data, accessed on 25 May 2024). However, the author’s permission through an agreement is required for academic purposes.

### 6.7. Dataset VII

Dataset VII [78] is collected using 10 LoRa devices using different SF values. The devices include LoPy4 and Dragino LoRa shields integrated with SX1276 chipsets. The frames were captured at 868.1 MHz and the BW was fixed at 125 kHz. On the receiving end, USRP N210 was placed at a distance of 0.5 m with a Sampling rate of 250 kHz. In total, 90,000 packets are collected from these devices whereas 75,000 of them are used for training. The remaining 15000 packets were separated for testing. The data were collected at a high SNR value of 70 dB hence it can be considered as noise-free data. This is the same dataset used in [73]. This dataset is publicly available and can be accessed at IEEE dataport (https://ieee-dataport.org/documents/lorarffidatasetdifferentspreadingfactors, accessed on 25 May 2024).

### 6.8. Dataset VIII

This dataset [77] was collected using 8 similar LoRa devices (model unknown). On the receiving end, the LoRa signals were captured using NI USRP-2942R SDR operating at a center frequency of 433 MHz with a bandwidth of 2 MHz. Each LoRa device was configured to send packets at the interval of 1 ms. Each file consists of 20,000 samples. The total size of the dataset is 0.8 GB. The dataset is not publicly available.

### 6.9. Dataset IX

Dataset IX is a comprehensive RFF dataset collected from real LoRa IoT devices under various environments [64]. The authors used 60 commercial off-the-shelf LoRa devices as transmitters and a USRP N210 software-defined radio platform as receivers for their experiments. The LoRa devices used for the experiment were chosen from different manufacturers, i.e., Pycom LoPy4, mbed SX1261 shield, Pycom Fipy, and Dragino SX1276. However, most of these devices, i.e., 45 were Pycom LoPy4. The USRP N210 was operated at 868.1 MHz with a spreading factor of 7 and a code rate of 4/8. The transmission interval between each frame was fixed to 0.3 s. The transmitting devices were placed at a distance of 0.5 m from the receiver. The dataset is available in multiple files categorized based on collection scenarios.

Additionally, the data are augmented to increase the number of samples and avoid the collection overhead. For data augmentation, several channel effects such as multipath fading, spreading, Doppler effect, and SNR are introduced in certain ranges. A separate file of the augmented data is also provided. For each device in the training data, 1000 frames are stored whereas for testing only 400 frames are stored. The data are stored in the complex128 data format into a Hierarchical Dataformat version 5 (HD5) file (https://www.hdfgroup.org/solutions/hdf5/, accessed on 25 May 2024). Each HD5 contains a preamble part of the LoRa signal with eight up-chirps converted consisting of 8192 samples. The dataset is publicly available for download at the IEEE data port (https://ieee-dataport.org/documents/lorarffidatasetdifferentspreadingfactors, accessed on 25 May 2024). The total size of the uncompressed dataset is 32 GB.

### 6.10. Dataset X

The dataset used in [75] is collected using 25 Pycom LoRa transceivers and 2 USRP B210 SDRs. Multiple datasets were collected under different configurations and environmental conditions to evaluate the proposed transformer model. The operating frequency was set as 915 MHz, where different SF options 7, 8, 11, 12 were considered. For all the configurations, the code rate was fixed at 4/5, and the sampling rate was fixed at 1 MS/s. The LoRa transceivers were placed in different indoor and outdoor environments. Each of the LoRa transmissions occurred for 20 s generating 20 million raw samples. The LoRa samples were stored as raw IQ using GNU radio. The dataset is not publicly available.

### 6.11. Dataset XI

Dataset XI discussed in [80] is captured using 10 MDots LoRa embedded nodes with SX1272 chipsets. All devices are from the same model and manufacturer, whereas BladeRF 2.0 SDR was used as a receiver operating at 915 MHz frequency and 2 MS/s sampling rate. The transmitters were configured to send LoRa signals at SF10 and 500 kHz BW. The data collection was conducted in an office environment with obstacles such as chairs and tables. The data were collected over three days to analyze the impact of time on signal variations. Each transmitter was rested for four hours before the next collection. The dataset is not publicly available.

### 6.12. Dataset XII

Dataset XII in [81] is collected using only eight LoRa devices including four Wifi-LoRa-32 boards and four Dragino shields mounted on Arduino boards. The HackRF SDR was used as a receiver to capture and save the LoRa RF signals in the GNU sink file. The data were collected in a 902–908 MHz frequency range with a bandwidth of 125 kHz. The authors do not provide any other necessary information such as spreading factor, sampling rate, or the nature or the duration of the capture. The dataset is publicly not available.

### 6.13. Dataset XIII

Dataset XIII in [84] is collected using 60 LoRa devices including 20 LoRa SX1278A kits from SENSORO and 40 kits from ASR Microelectronics. The Ettus Research B205 USRP SDR was used as a receiver to capture and save the LoRa RF signals. Except for the bandwidth value (125 kHz), no further information is detailed by the authors in the paper. The data were gathered under different scenarios including indoor LOS, NLOS, and outdoor LOS at a distance of 100 m. In addition, a dynamic scenario was also created where the LoRa transmitter will move around the USRP in a circular path. The dataset is also not publicly available.

### 6.14. Lessons Learned

**Figure 12:**
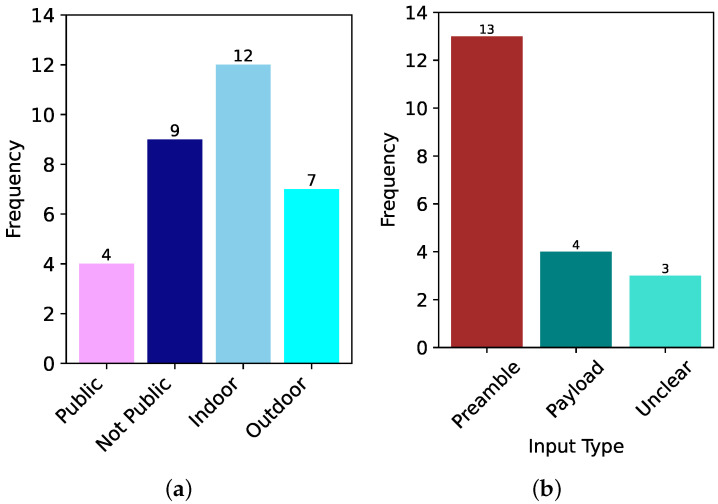
Dataset information: data collection environment, availability, and the type of data type used to train models (**a**) Existing LoRa datasets (**b**) Data type used to train the model.

Publicly available datasets help the research community to experiment with the data and propose solutions. LoRa RFFI systems require massive and diverse data for training. However, we have shown that only a few datasets are publicly available. As can be seen in Figure 12a, only four datasets are publicly accessible. Among these four, Dataset VI used in [68] is not openly available. We contacted the authors and signed the agreement for research purposes but did not receive any response. Additionally, the quality of the publicly available datasets is also questionable. Moreover, they are collected in a mostly controlled laboratory environment (indoor and outdoor). It is vital to collect LoRa RFF data in all possible environments with various combinations of LoRa parameters which can act as a benchmark for the community. It is also important to look at the format of the data used for training the model. It can be observed from Figure 12b that the majority of the time (13 times), the DL models were trained using only the preamble part of the LoRa frame. Only for four instances, the models were trained using the payload as well. Although, it is not recommended to use payload as the DL model can be based on the payload information.

**Figure 13:**
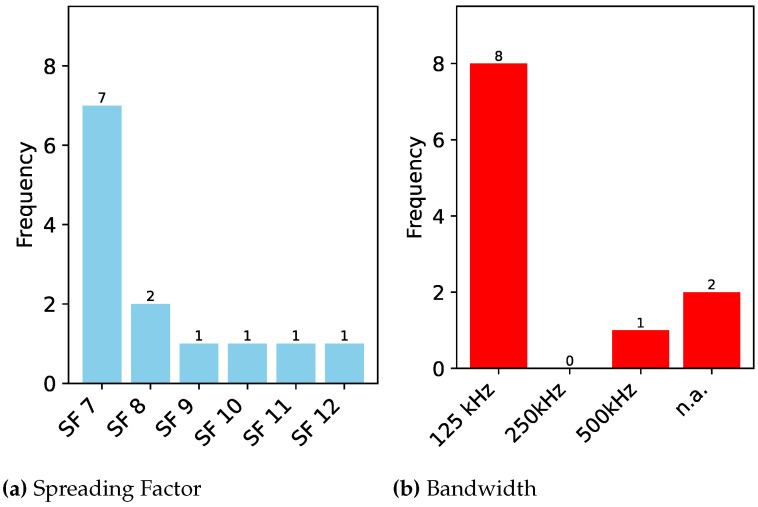
Frequency of LoRa parameters.

It is also observed that the majority of the collected LoRa signal was transmitted using a spreading factor of 7 and a bandwidth of 125 kHz, as shown in Figure 13. It is important to evaluate the models on the varying range of LoRa parameters as they impact the waveform shape and duration, as well as the number of samples. These factors can affect the performance of the deep learning model.

While majority of the studies focus on using only the preamble part of the signal which is the correct way to train a deep learning algorithm. It has been observed that some of the studies also use the payload part of the LoRa frame [67,68]. This introduces a bias for the model as it presents the easy-to-remember information: the size and the content of the payload. In that case, it is important to either change the content or the byte size used, otherwise, the model will provide high accuracy in one case but cannot generalize well on data with different sizes and content.

## 7. Challenges and Future Research Directions

**Figure 14:**
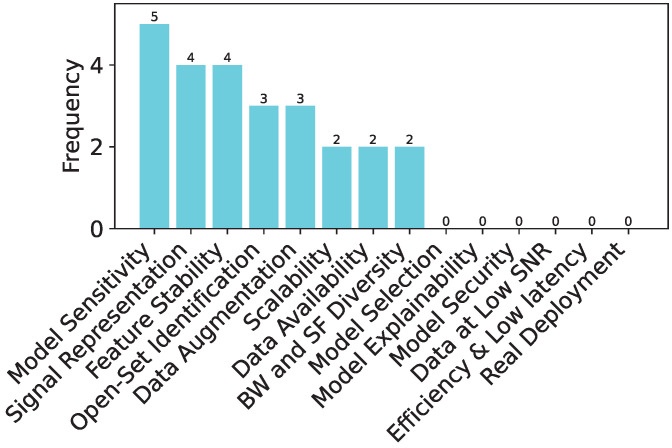
Challenges addressed.

After reviewing the existing literature in the field of deep learning-based LoRa radio frequency fingerprinting (RFFI), it becomes clear that several research challenges need to be addressed. A summary of those challenges is presented in Figure 14 along with a count of the papers that deal with or partially address some of them. In this section, we aim to elucidate all the research challenges derived from the current state of the art. We divide these challenges into general and LoRa-specific.

### 7.1. General DL-Based RFFI Challenges

#### 7.1.1. Data Capture at Low SNR

Most research studies on LoRa RFFI are based on high SNR signals [73,78]. However, one of the main benefits of LoRa is its ability to demodulate signals at very low and even negative SNRs. For example, using SF 12, Semtech claims the ability to demodulate with an SNR as low as −20 dB. It can therefore be expected that many real-world LoRa signals will have low SNR values. How DL-based RFFI systems behave with low SNR remains to be thoroughly investigated.

#### 7.1.2. Feature Stability

Deep learning-based LoRa RFFI systems learn patterns from device-specific features such as CFO, SFO, and IQ imbalances. However, these features are affected by changes in the environment, especially by temperature variations, hence resulting in a different behavior than the original used for training. This change in behavior over time can severely affect the performance of the DL model. Therefore, devising or selecting stable features is critical for the long-term operation of the system.

#### 7.1.3. Scalability

At the implementation level, scalability is a major challenge. Currently, the work in this domain is in the laboratory stages and is being experimented with a limited number of transmitters. In reality, there will be a massive number of devices and the number will increase with time. Hence, the LoRa RFFI system must be capable of meeting the scalability requirements. Therefore, working in this direction is also a key challenge that the research community needs to look at.

#### 7.1.4. Data Augmentation

DL models are severely affected by the channel effects entangled with the signal at the receiver side. If the channel effect is learned by the deep learning model, its ability to identify known devices deteriorates with changes in the channel behavior. To improve the robustness of the DL models, data augmentation has been incorporated by some authors. For example, the training signal may first be passed through a noisy signal with fading effects [64,68]. Data augmentation during training helps in increasing the data samples and can also provide signals with different SNR values, resulting in improved model robustness. Choosing adequate data augmentation is a challenging task that requires further attention.

#### 7.1.5. Model Sensitivity

We have already mentioned that the DL models are sensitive to changes in the environment (Section 7.1.4), parameters (Section 7.2.2), hardware (Section 7.1.2), and signal representation (Section 7.2.3). Hence, devising a model that works equally well in all situations is a key challenge to handle in a DL-based LoRa RFFI system. Some of the researchers have proposed domain or channel-agnostic solutions for this problem [64,75] but with limited success. Hence, it is recommended to look further into this direction.

#### 7.1.6. Model Security

DL models are sensitive to data perturbations and it is shown that they can be cracked with adversarial attacks [100]. Hence, the security of DL models itself is also important which has been completely overlooked in this domain. We believe it is also a future challenge that requires attention.

#### 7.1.7. Model Explainability

The DL models are like black boxes and we do not know how the decisions are made from the data. Similar to the other fields, DL models for LoRa RFFI learn from signals and predict output in a closed manner. We do not know what LoRa signal feature contributed more towards accurate identification of the devices. Hence, model explainability can also be seen as a future challenge.

### 7.2. LoRa-Specific Challenges

#### 7.2.1. Data Availability

Properly capturing LoRa frames for RFF is challenging, due to the need for environmental, parametric, and temporal diversity in the scenarios. Sharing the outcome of such efforts with the community is therefore of tremendous importance. In Section 6, we highlighted and briefly described all the datasets that are used for LoRa device identification using radio frequency fingerprinting. However, only a small subset of them are publicly accessible [47,64,68]. For any deep learning-based problems, the availability of significant quality data is crucial. Furthermore, from a scientific point of view, it would be preferable to agree on a set of public data enabling all the proposed algorithms to be evaluated on a common basis.

#### 7.2.2. BW and SF Diversity

LoRa CSS modulation depends on two main parameters: the bandwidth (BW) and the spreading factor (SF). These parameters are typically selected by the end device firmware, possibly under the control of the LoRaWAN servers, using the ADR mechanism. A change in bandwidth or spreading factor results in different chirp durations and signal waveforms, as described in Section 2.3. This has important consequences in terms of recognizing an end device using a deep learning model. Indeed, depending on these parameters, the same payload will be carried by signals of different shapes and lengths while models such as MLP and CNN lack the capability to deal with variable-size input signals. Despite this, we discovered that the majority of existing research primarily concentrates on identifying LoRa signals based on fixed-length packets based on particular BW/SF pairs, as summarized in Figure 13. Very few research has approached this challenge using, e.g., RNNs and transformer models [73,78]. The impact of variations in these parameters needs to be systematically studied.

#### 7.2.3. Signal Representation

LoRa signals can be represented in different forms such as IQ, (A/ϕ), FFT, and spectrograms (Section 4.3), IQ and spectrograms being the most used representations (Figure 11b). Moreover, most recent studies have turned to the use of spectrograms which are considered the most appropriate for LoRa device identification. However, there is still room for study in this area to investigate additional signal transforms such as, e.g., wavelets or Hilbert that could help deep learning models to learn the end-device-specific hardware impairments better.

#### 7.2.4. Model Selection

Many different deep learning models have been used in the literature reviewed. The rationale for using a particular model for LoRa RFFI is generally not provided. As a result, we found that most studies simply select models at random and rely on a trial-and-error strategy. More importantly, although some studies claim that one model is better than another, we do not have a common basis for comparing algorithms. In conclusion, the selection of an appropriate deep learning algorithm remains one of the main challenges.

#### 7.2.5. Open-Set Identification

Most studies focus on the closed-set identification of LoRa devices. This means only devices available during the training set will be identified. Hence, for any new device to be included in the network will require retraining of the model which is not the ideal scenario in the real world. Therefore, open-set identification for the LoRa RFFI system is still a challenge that is worth solving.

#### 7.2.6. Efficiency and Low Latency

DL-based RFFI systems are meant to be used in real-time, as a complement to the regular cryptography-based authentication. Hence, bounding their latency is of utmost importance. Moreover, given the range of possible DL models and LoRa signal representations, there is interest in exploring the trade-off between their number of parameters, their need for computational resources and the performance they are able to achieve.

#### 7.2.7. Real Environment Deployment

The existing studies have only focused on data collection at laboratory test bed scenarios with limited devices and a controlled environment. As far as we know, no deployment in a real-world operational LoRaWAN network has been reported in the literature. The actual efficacy of the LoRa RFFI system can be measured only in a real environment.

### 7.3. Future Research Directions

#### 7.3.1. Transfer Learning

Transfer learning is the idea of using an already-existing, pre-trained deep learning model for a particular problem. It offers multiple advantages, such as reduced time and resource consumption for training the model from scratch, effective regularization, and improved performance. It has not yet been used intensively; hence, we recommend investigation into transfer learning for the LoRa RFFI system in future research works.

#### 7.3.2. Impact of Receiver Imperfections

Most existing research works emphasize transmitter imperfections. However, the SDR can also have impairments, which can also be taken into account for LoRa RFFI systems. Not many researchers have paid attention to this area except [47,75]. LoRa(WAN) networks are expected to cover large areas, hence there will be a need to capture the LoRa RF signal from different vantage points that is with multiple receivers. One can even imagine that in the future, every gateway might act as an SDR. The RFFI system will then need to get “test” data from multiple receivers, while the DL model is presumably trained only with data collected from a single vantage point. This makes the receiver’s imperfections more relevant.

#### 7.3.3. Proper Modeling of Device Imperfections

For accurate and stable identification of LoRa devices using hardware imperfections, it is vital to properly model those imperfections. It is important to recall that all the studies assume a direct-conversion receiver architecture while a different architecture might be used in actual LoRa transceivers. This different architecture comes with its specific set of hardware impairments, some of them possibly different from those of the DC architecture as indicated in Figure 8. Moreover, given that, to the best of our knowledge, all the transceivers are manufactured by Semtech (Camarillo, CA, USA), all devices might use that architecture, which makes this question particularly important.

#### 7.3.4. Universal Set of RFF Features

The existing RFF systems rely on particular characteristics of the wireless communication technology, such as modulation parameters. Therefore, it remains a protocol-specific RFF system. Creating a universal set of features that remains applicable to all kinds of RF devices and protocols is also an area of open research.

#### 7.3.5. LoRa Operating at 2.4 GHz

Announced in 2017 by Semtech, LoRa at 2.4 GHz is now a reality. Surprisingly, to the best of our knowledge, no study has been published on its specific impairments. The 2.4 GHz ISM band is one of the most crowded ones. This means that an RFFI system working in this band will face lower SNR signals. Moreover, the range of LoRa spreading factors has been extending down to SF 5 while the channels can be as wide as 1625 kHz. With these extreme values, the duration of a chirp becomes shorter than 20 μs. This means that a higher sampling rate might be required. We believe studying how DL-based LoRa RFFI systems will fare for this version of LoRa is an interesting research direction.

## 8. Conclusions

In this paper, we present a comprehensive and structured survey of the state of the art in the domain of deep learning (DL) based LoRa radio frequency fingerprinting (RFFI) systems. To this end, we have reviewed the literature on the selected topic over the last decade. We discuss various aspects of a DL-based RFFI and present our analysis of these aspects. We first recall the necessary details about the LoRa technology and the main RF transmitter hardware impairments that can be visible in the RF signal and used as fingerprints. We then examine several categories of RF fingerprinting approaches, including those based on deep learning.

We provide an in-depth explanation of a typical DL-based LoRa RFFI architecture to outline its successive steps, possible signal representations, design choices, and trade-offs. With this vision in mind, we proceed to a systematic description of the reviewed literature, which we have grouped according to the main deep learning techniques used. Our analysis highlights the author’s justifications behind their specific deep learning architecture, their evaluation methodology, and the results obtained. In addition, we reviewed all the LoRa RFFI datasets discussed in the literature and provided a tabular summary. We also briefly present the type of hardware and the parameters used in the data collection process. Finally, we contribute a detailed discussion of various challenges that we believe remain open in this area and indicate some future directions for research.

One of the main conclusions of our survey is that there is clear interest in RFFI and that partial success has been achieved, although real-world use is limited. There are many research opportunities that can be exploited to further enhance the performance of LoRa RFFI systems. As an example, it is very important to have datasets on which the scientific community has agreed to benchmark the proposed systems. The same goes for the ability to understand which RF signal features contribute the most to how a deep learning model is able to identify a device.

## Figures and Tables

**Table 1:** DL models for LoRa RFFI.

Year	Reference	MLP	CNN	LSTM	ResNet	Transformers	Hybrid
2017	Robyns et al. [67]	✔	✔				
2018	Das et al. [72]			✔			
2019	Jiang et al. [69]	✔	✔				
2021	Elmaghbub et al. [47]		✔				
2021	Shen et al. [53]		✔				✔
2021	Al-shawabka et al. [68]		✔	✔			
2021	Shen et al. [65]	✔	✔	✔			
2021	Shen et al. [73]					✔	
2021	Zhang et al. [74]		✔				
2022	Shen et al. [64]				✔		✔
2022	Gaskin et al. [75]						✔
2022	Zhang et al. [76]		✔		✔		✔
2022	Qi et al. [77]		✔				
2023	Shen et al. [78]		✔	✔		✔	
2023	Qi et al. [79]				✔		✔
2023	Gao et al. [80]		✔				
2023	Mex-Perera et al. [81]		✔				
2024	Shen et al. [82]		✔				
2024	Baldini et al. [83]		✔				
2024	Guo et al. [84]		✔				
2024	Ahmed et al. [85]		✔		✔		

**Table 2:** Summary of the existing literature on DL-based LoRa RFFI.

Year	Reference	Main Approach	Signal Representation	Remarks
2017	Robyns et al. [67]	Complete LoRa frame with payload bytes is used for RFF. The DL models (MLP and CNN) are trained per the symbol of the frame.	IQ	CFO effect is not considered which affects the performance of the RFFI system.
2018	Das et al. [72]	Lightweight LSTM-based LoRa RFFI system is proposed. LSTM with 1, 2, and 3 layers are compared on the same LoRa IQ dataset.	IQ	One layer LSTM outperformed the deeper LSTM models. Performance degraded under low SNR values.
2019	Jiang et al. [69]	Differential constellation trace figure is created from LoRa IQ signal and a Euclidean distance-based clustering method is proposed to identify a LoRa device.	Constellation figure	The method is tested with only 6 LoRa devices. The authors leave the evaluation of scalability as future work.
2021	Elmaghbub et al. [47]	Out-of-band distortion caused by hardware components were exploited for LoRa device identification using CNN.	IQ, FFT, (A/ϕ)	Does not perform under different scenarios (days, configuration, time, receiver).
2021	Shen et al. [53]	CFO estimation and compensation is shown as a crucial element in the RFFI system for LoRa. CNN is used as a classifier using a spectrogram generated from LoRa IQ signals.	IQ, FFT, Spectrogram, CFO	CFO is an unstable feature for RFFI systems, therefore, compensating CFO is important for accurate device identification using RFF.
2021	Al-shawabka et al. [68]	A data augmentation technique is introduced to enhance the robustness of LoRa RFFI systems.	IQ, Amplitude/phase (A/ϕ), Spectrogram	The efficiency of the proposed method is affected by changes in the environment.
2021	Shen et al. [65]	Effect of CFO variation on DL-based RFFI system is studied using IQ, FFT, and spectrogram. A hybrid CNN model outperforms other models	IQ, FFT, Spectrogram, CFO	CFO calibration and compensation are mandatory for the RFFI system.
2021	Shen et al. [73]	Transformer-based LoRa RFFI system is proposed for LoRa signal with different SF. The effect of different augmentation methods is studied.	IQ, CFO	The proposed method is only good for high SNR values.
2021	Zhang et al. [74]	PA non-linearity and IQ imbalance are used to train and test a CNN model.	IQ, PA non-linearity, IQ imbalance	Performance degrades when using a different receiver.
2022	Shen et al. [64]	A ResNet-based extractor model is used with deep metric learning and *k-NN* as a classifier. The proposed RFFI system consists of three stages: feature extraction, enrollment, and identification.	Spectrogram	The open-set recognition system varies in performance if tested under different environments.
2022	Gaskin et al. [75]	A CNN-based hybrid approach called Tweak is introduced for the domain-agnostic LoRa RFFI system. A multi-receiver scenario is presented.	IQ	The performance is degraded under varying environmental conditions.
2022	Zhang et al. [76]	A hybrid model is proposed to extract RFF features hidden in non-stationary LoRa signal. It uses linear translation-variant multiscale fractional wavelet filters to reduce the influence of noise on feature extraction.	Spectrogram, Fractional Wavelet Transform	The further transformation of the LoRa signal from IQ can improve the performance of DL models.
2022	Qi et al. [77]	Amplitude/Phase and statistical features are used as input to train a CNN model.	IQ, (A/ϕ), statistical features	The work lacks discussion on the scalability of the proposed method.
2023	Shen et al. [78]	Several models are proposed including CNN, LSTM, GRU, and transformer for the task of LoRa device classification.	Spectrogram	Only 10 LoRa devices were used for data collection. The scalability of such a model is a challenging issue.
2023	Qi et al. [79]	An ensemble of ResNet34, Inceptionv3, and DenseNet121 is presented for LoRa device identification using RFF.	Spectrogram	The ensemble model provides better accuracy than the individual baseline models.
2023	Gao et al. [80]	Spectrogram merged with CFO are used as input to train a CNN model which enhances the robustness of the proposed technique.	Spectrogram, CFO	The model achieves higher accuracy than the state of the art.
2023	Mex-Perera et al. [81]	A lightweight CNN architecture evaluated using raw IQ signals and IQ imbalanced data.	IQ imbalance	The method requires further evaluation using a larger dataset and diverse environments.
2024	Shen et al. [82]	Open set identification solution is provided using metric learning and fine-tuning.	Spectrogram	A multi-location scenario is tested where the proposed solution achieves over 90% accuracy.
2024	Baldini et al. [83]	A novel method called VMD is presented to learn intrinsic device features in spectrogram using DL.	Spectrogram	Tested on only 10 devices.
2024	Guo et al. [84]	A novel RFF extraction method based on cyclic shift property of LoRa CSS modulation is presented	PSD	The method performs better in different environments compared to some existing studies.
2024	Ahmed et al. [85]	Existing complex model is optimized into low complexity.	Spectrogram, IQ, FFT, A/ϕ	The complexity and accuracy trade-off can further be evaluated on large scale dataset.

**Table 3:** Detailed summary of DL model architectures used in the literature.

Year	Reference	Models	Number of Layers	Activation	Optimizer	Code Availability
2017	[67]	CNN	2, Conv1D, 1 FC, Softmax	ReLU	NA	No
2018	[72]	LSTM	1-2 Layers	NA	NA	No
2019	[69]	CNN	NA	NA	NA	No
2021	[47]	CNN	6, Conv2D, 2 Fc, Softmax	LeakyReLU	SGD	No
2021	[53]	CNN	2, Conv2D, 2 Fc, Softmax	ReLU	Adam	No
2021	[68]	CNN	2, Conv1D, 3 Fc, Softmax	ReLU	NA	
		CNN	2, Conv2D, 3 Fc, Softmax	ReLU	NA	No
		LSTM	3 LSTM, 1 Fc, Softmax	ReLU	NA	
2021	[65]	CNN	3 Conv2D, 1 Fc, Softmax	ReLU	Adam	
		LSTM	2 LSTM, 1 Fc, Softmax	ReLU	Adam	No
2021	[73]	Transformer	2 sub-blocks, 1 Fc, Softmax	NA	NA	No
2021	[74]	CNN	5 Conv2D, 2 Fc, Softmax	ReLU	Adam	
2022	[64]	ResNet	9 Conv2D, 1 Fc	ReLU	RMSprop	Yes
2022	[75]	Siamese Network	2 Pairs of CNN, Triplet Loss	NA	NA	No
2022	[76]	DFSNet Hybrid	Multiple layers for each ResNet, AlexNet, and CNN	ReLU	NA	No
2022	[77]	CNN Hybrid	Multiple layers for each ResNet34, Inception, and DenseNet	NA	NA	No
2023	[78]	ResNet	10, conv2D, 1 Fc, Softmax	ReLU	NA	
		LSTM	2 LSTM, 1 Fc, Softmax	ReLU	NA	No
		GRU	2, GRU, 1 Fc, Softmax	ReLU	NA	
		Transformer	4 sub-blocks, 1 Fc, Softmax	NA	NA	
2023	[79]	CNN	1 Conv2D, 2 Fc, Softmax	NA	SGD	
2023	[80]	CNN	6 conv1D, 1 Fc, Softmax	ReLU	Adam	No
2023	[81]	CNN	2 conv2D, 2 Fc, Softmax	ReLU	Adam	No
2024	[82]	CNN	3 Conv2D, 1 Fc, Softmax	ReLU	Adam	No
2024	[83]	CNN	2 Conv2D, 1 Fc, Softmax	ReLU	Adam	No
2024	[84]	CNN	4 Conv1D, 1 Fc, Softmax	ReLU	AdaDelta	No
2024	[85]	ResNet	5 Conv2D, 1 Fc, Softmax	ReLU	RMSprop	No
		CNN	3 Conv1D, 2 Fc, Softmax	ReLU	RMSprop	No

Legend: 1D-CNN: Conv1D, 2D-CNN: Conv2D, Fully Connected (Fc), Stochastic Gradient Descent: (SGD).

**Table 4:** Summary of performance metrics across different models. note that the accuracy reported here is the best-case scenario in each of the works.

Year	Reference	Signal Representation	Model	Best Accuracy (%)	Comments
2017	[67]	IQ	CNN	98.00	Same dataset (train-test)
2018	[72]	IQ	LSTM	99.58	2 layer LSTM (<1 m distance)
2019	[69]	DCTF	CNN, Clustering, MLP	99.00	99% at 10 MS/s and 5 m.
2021	[47]	IQ, FFT, Amp/Phase	CNN	95.00	Using FFT at same location data (train-test)
2021	[53]	IQ, FFT, Spectrogram	CNN, Hybrid	97.61	Using Spectrogram 92% IQ, 92.31% with FFT.
2021	[68]	IQ, A/ϕ, Spectrogram	CNN, LSTM	82.00	Using 100 devices on a different day data.
2021	[65]	IQ, FFT, Spectrogram	CNN, LSTM, MLP, Hybrid	98.11	Using IQ, 98.11, FFT, 85.58%, Spectrogram, 96.40.
2021	[73]	Spectrogram	Transformer	99.00	99% with 10 devices at 30 dB.
2021	[74]	IQ	CNN	99.00	99.00% on same data payload. Varying accuracy was achieved for different scenarios.
2022	[64]	Spectrogram	CNN Hybrid	98.50	98.50% in one the scenario for other scenarios the accuracy degrades.
2022	[75]	IQ	CNN	Not Specified	The authors use Area Under the Curve (AUC) as an evaluation metric.
2022	[76]	Spectrogram	CNN Hybrid	98.50	98.5% is on DFSNet, while different models were used ResNet.
2022	[77]	IQ	CNN	93.25	Limited dataset used.
2023	[78]	Spectrogram	CNN, LSTM, GRU, Transformer	99.99	99.99% with CNN on augmented data. It is difficult to point out one accuracy as it is presented for all models with different aspects.
2023	[79]	Spectrogram	Hybrid model (ResNET, inception, denseNet)	95.10	Ensemble model achieves better accuracy than individual models used in the study.
2023	[80]	Spectrogram	CNN	99.50	The best accuracy is achieved on the same day. For a different day the accuracy is reduced.
2023	[81]	IQ	CNN	97.00	Only 8 devices used. Lack of robustness.
2024	[82]	Spectrogram	CNN	90.00	Over 90% accuracy but with high SNR of 35 dB.
2024	[83]	Spectrogram	CNN	91.48	This accuracy is achieved with a window length of 256 and VMD residuals.
2024	[84]	PSD	CNN	98.42	A novel RFF method using PSD is introduced.
2024	[85]	Spectrogram, IQ, FFT, A/ϕ	ResNet	97.42	The complexity performance trade-off can be evaluated on the large-scale datasets.

**Table 5:** Summary of LoRa RFFI datasets.

Dataset	Year	Reference	Type	Number of Devices	Public	Environment		Size	LoRa Parameters			
						**Indoor**	**Outdoor**		**Fc (MHz)**	**BW (kHz)**	**SF (MS/s)**	**SR**
I	2017	[67]	IQ	22	✔	✔	-	-	868.1	-	7	1
II	2019	[69]	IQ	6	-	✔	-	-	433	125	7	5
III	2021	[47]	IQ, FFT	25	✔	✔	✔	1.2 TB	915	125	7	1
IV	2021	[65]	IQ	25	-	✔	-	-	868.1	125	7	1
V	2021	[74]	IQ	5	-	✔	✔	-	868.1	125	7	1
VI	2021	[68]	IQ	100	✔	✔	✔	1 TB	902.3	-	-	-
VII	2021	[73]	IQ	10	✔	✔	✔	90,000 frames	868.1	125	7, 8, 9	0.25
VIII	2022	[77]	IQ	8	-	-	0.8 GB	433	-	-	-	-
IX	2022	[64]	IQ	60	✔	✔	✔	32 GB	868.1	125	7	1
X	2022	[75]	IQ	25	-	✔	✔	-	915	125	7, 8, 11, 12	1
XI	2023	[80]	IQ	10	-	✔	-	-	915	500	10	2
XII	2023	[81]	IQ	8	-	✔	-	-	-	125	-	-
XIII	2024	[84]	IQ	60	-	✔	✔	35,870 frames	-	125	-	-

Legend: Carrier frequency (Fc), Channel Bandwidth (BW), Spreading Factor (SF), Sampling Rate (SR), Not Available (-).
